# Late Effects in Survivors of Adolescent and Young Adult Acute Lymphoblastic Leukemia

**DOI:** 10.1093/jncics/pkaa025

**Published:** 2020-04-02

**Authors:** Lori Muffly, Frances B Maguire, Qian Li, Vanessa Kennedy, Theresa H Keegan

**Affiliations:** p1 Division of Blood and Marrow Transplantation, Department of Medicine, Stanford University, Medical Center, Stanford, CA, USA; p2 California Cancer Reporting and Epidemiologic Surveillance Program, University of California Davis Health, Institute for Population Health Improvement, Sacramento, CA, USA; p3 Division of Hematology and Oncology, Center for Oncology Hematology Outcomes Research and Training (COHORT), University of California, Davis School of Medicine, Sacramento, CA, USA; p4 Department of Medicine, University of California, San Francisco, CA, USA

## Abstract

**Background:**

Knowledge regarding late effects (medical conditions and subsequent neoplasms) in survivors of adolescent and young adult (AYA) acute lymphoblastic leukemia (ALL) is lacking.

**Methods:**

Using the population-based California Cancer Registry linked with California hospitalization data, we evaluated late effects in 1069 AYAs (aged 15–39 years) diagnosed with ALL in California between 1995 and 2012 and surviving a minimum of 3 years from diagnosis.

**Results:**

The estimated 10-year cumulative incidence of subsequent endocrine disease (28.7%, 95% confidence interval [CI] = 25.8% to 31.6%) and cardiac disease (17.0%, 95% CI = 14.6% to 19.5%) were strikingly high; avascular necrosis (9.6%, 95% CI = 7.8% to 11.6%), liver disease (6.5%, 95% CI = 5.0% to 8.3%), respiratory disease (6.2%, 95% CI = 4.8% to 8.0%), seizure and/or stroke (4.3%, 95% CI = 3.1% to 5.8%), renal disease (3.1%, 95% CI = 2.1% to 4.4%), and second neoplasms (1.4%, 95% CI = 0.7% to 2.4%) were estimated to occur at 10 years with the reported frequencies. Multivariable analyses including the entire patient cohort demonstrated that public or no insurance (vs private and/or military insurance) and receipt of hematopoietic cell transplantation were independently associated with the occurrence of all late effects considered. In multivariable analyses limited to the 766 AYAs who were not transplanted, we continued to find a statistically significant association between public and no insurance and the occurrence of all late effects. Frontline regimen type (pediatric vs adult) was not statistically significantly associated with any of the late effect categories.

**Conclusions:**

This large population-based analysis is among the first to describe late effects in survivors of AYA ALL. The strong association between insurance type and late effects suggests that AYAs with public or no insurance may have reduced access to survivorship care following completion of ALL therapy.

As the survival of adolescents and young adults (AYAs) with acute lymphoblastic leukemia (ALL) improves, a greater focus on late effects and survivorship issues is increasingly necessary. The medical conditions and subsequent neoplasms (hereafter referred to as late effects) that develop following the successful treatment of ALL in children have been captured and described through a variety of registries, including the Childhood Cancer Survivor Study (CCSS) (1–4). No such data source exists for survivors of young adult cancers, and late effects data regarding ALL patients diagnosed and treated in adult cancer settings have been particularly scarce (5).

The treatment landscape of AYA ALL has evolved over the past 2 decades. Although novel targeted immunotherapies and cellular therapies are now routinely used in relapsed or refractory ALL, multiagent chemotherapy remains the backbone of AYA ALL treatment (6). AYA ALL patients have historically received either pediatric or adult frontline ALL regimens depending on where they obtain their cancer treatment, clinical trial availability, and preference and expertise of the treating team (7). Traditional adult ALL regimens incorporate more cytotoxic, myelosuppressive agents, whereas pediatric regimens include a greater use of steroids, asparaginase, vincristine, and intrathecal therapies. These distinctions led some to posit that the development of late effects may differ in AYA ALL patients depending on whether they received a pediatric or adult ALL regimen (8).

To begin to fill these gaps in current knowledge, we conducted an observational study evaluating late effects in survivors of AYA ALL across the state of California. Using population-based data, we additionally describe associations between late effects and AYA sociodemographics and ALL therapies, including frontline regimen type, in a large cohort of AYA ALL survivors.

## Methods

### Data Sources

We identified patients aged 15 to 39 years at the time of ALL diagnosis between January 1, 1995, and December 31, 2012, in the California Cancer Registry (CCR) using the *International Classification of Diseases* (*ICD*) *for Oncology*, 3rd edition, codes 9826, 9835, 9836, 9811-9818, and 9837. The CCR is California’s population-based cancer surveillance system, which is composed of 3 National Cancer Institute Surveillance Epidemiology and End Results registries. The CCR has been state mandated to collect reports on incident cancers diagnosed in California since 1988. Patients surviving less than 3 years from diagnosis were excluded from the study cohort.

**Figure 1.**
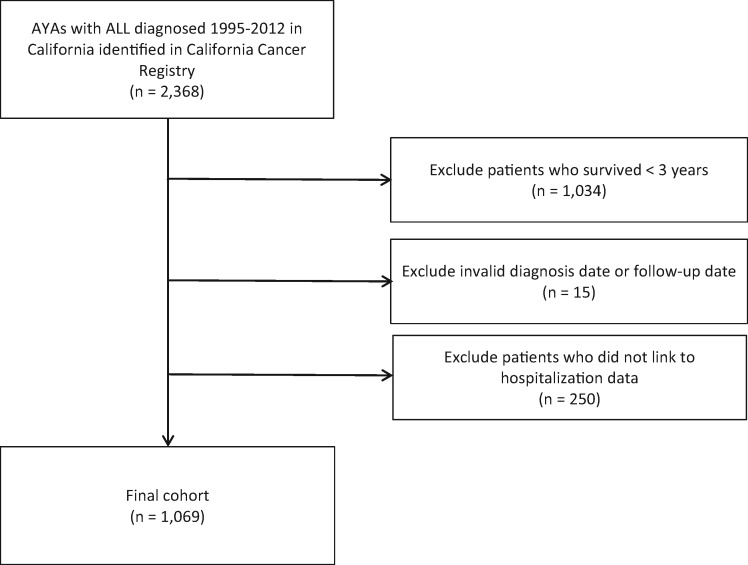
Study cohort diagram. ALL = acute lymphoblastic leukemia; AYA = adolescent and young adult.

Identified patients were linked to the Office of Statewide Health Planning and Development (OSHPD) patient hospital discharge records through a probabilistic linkage that matched on social security number, date of birth, sex, and zip code. Patients who did not link to the OSHPD file (n = 250, of which 203 were unable to link because of invalid social security numbers) were excluded (*see*Figure 1). OSHPD contains longitudinal information for each patient on all admissions at nonfederal (eg, not military or Veterans Administration) acute care hospitals in California and includes information on principal hospital diagnosis and up to 24 secondary diagnoses based on the *ICD*, *9th* *Revision* (*ICD-9*) and the *ICD*, *10th Revision* (*ICD-10*).

We obtained patient characteristics and treatment information from CCR including age at diagnosis, sex, race and ethnicity, health insurance at diagnosis or initial treatment, neighborhood socioeconomic status (SES) tertile, treatment facility, physician specialty (pediatric vs adult oncologist), cranial irradiation treatment (CRT), and frontline regimen type. We considered publicly insured and uninsured patients together in the analyses because prior evaluations demonstrated that most AYAs who obtain public insurance at diagnosis were either uninsured or had intermittent public insurance just prior to diagnosis, and therefore the small number of uninsured AYA cancer patients in the cancer registry likely reflects retroactive enrollment in Medicaid at cancer diagnosis (9,10). Private health insurance included health maintenance organizations, preferred provider organizations, and military insurance. Neighborhood SES incorporates census block group-level data on income, education, housing costs, and employment (11,12). 

We determined CRT and frontline regimen type by manually reviewing treatment text fields in the CCR data, as described previously (7,13). We classified patients with missing or unknown frontline regimen (40.9%, n = 437) in the text fields using an algorithm based on their age and oncologist specialty (pediatric vs adult) as follows: pediatric regimen (aged 18 years and younger and/or 19-29 years with a pediatric oncologist), adult regimen (aged 19-39 years with an adult oncologist and treated prior to 2008), unclear (aged 19-39 years with an adult oncologist and treated after 2008). Hematopoietic cell transplant (HCT) and late effects were determined from OSHPD *ICD-9* and *ICD-10* discharge diagnosis codes (Supplementary Table 1, available online), whereas second neoplasms (SN) were identified in CCR data. Late effects were included if documented at 3 or more years following diagnosis and categorized as follows: cardiac (hypertension, coronary disease, cardiomyopathy and/or congestive heart failure), seizure and/or stroke, respiratory (asthma, chronic obstructive pulmonary disease, pulmonary fibrosis, pneumonopathy), renal (hypertensive renal disease, chronic kidney disease, kidney transplant, hemodialysis), liver (chronic liver disease, cirrhosis, liver transplant, chronic pancreatitis), endocrine (hypothyroidism, thyroid disease, diabetes mellitus, other disease of endocrine glands), and avascular necrosis (AVN).

### Statistical Analysis

Descriptive statistics (frequencies, percentages) characterized baseline patient, hospital, and treatment attributes. We used nonparametric methods that account for death as a competing risk to calculate cumulative incidence (CMI) and associated 95% confidence intervals (CIs) of developing a late effect 3 or more years after diagnosis (14). For CMI, the time to event was calculated starting from 3 years after cancer diagnosis until the first diagnosis of the given late effect; patients without the given late effect were censored on the date of last known contact or date of study cutoff (December 31, 2015), whichever occurred first.

We used Gray’s K-sample test statistic to determine if CMI of late effects differed by baseline characteristics over the entire study period (15). We used multivariable Cox proportional hazards regression to calculate adjusted hazard ratios (HRs) and 95% confidence intervals to evaluate sociodemographic and clinical characteristics associated with the occurrence of each late effect, defining late effect as an event occurring at least 3 years after diagnosis. Effect modification was assessed between HCT and health insurance by including interaction terms in the multivariable models. No interaction terms were statistically significant at a *P* value of less than .05. The proportional hazards assumption was assessed numerically based on cumulative sums of Martingale residuals and visually by plotting the log of negative log of the Kaplan-Meier estimates vs the log of time for all predictor variables. Variables that violated this assumption (treatment facility, age group) were included as stratifying variables. Correlation of variables in the models was assessed using chi-square tests. Multivariable models for late effects were adjusted for race and ethnicity, sex, health insurance, diagnosis year, neighborhood SES, and radiation. All models (*see*Tables 3–5) were stratified by age group and location of care, which violated proportional hazards assumptions. Frontline regimen type and HCT were highly correlated (*P* < .001); thus, models in the entire cohort included transplant, but not frontline, regimen. We also evaluated late effects separately in patients who did not undergo transplant (*see*Table 4) and who were transplanted (*see*Table 5) and included frontline regimen.

**Table 3.** Multivariable-adjusted HR and associated 95% CI of late effects among 3-year AYA ALL survivors, California, 1995-2012, including the entire study cohort (n = 1069 in each model)*

Variable	Cardiac diseases	Seizure/Stroke	Respiratory system diseases	Renal disease	Liver disease	Endocrine disease	Avascular events	Second cancers
	HR (95% CI)	HR (95% CI)	HR (95% CI)	HR (95% CI)	HR (95% CI)	HR (95% CI)	HR (95% CI)	HR (95% CI)
Race/ethnicity								
NH white	1.00 (Referent)	1.00 (Referent)	1.00 (Referent)	1.00 (Referent)	1.00 (Referent)	1.00 (Referent)	1.00 (Referent)	1.00 (Referent)
Hispanic	0.95	1.09	1.07	1.09	1.23	0.97	1.17	1.15
	(0.72 to 1.27)	(0.79 to 1.51)	(0.78 to 1.46)	(0.79 to 1.51)	(0.89 to 1.70)	(0.75 to 1.27)	(0.87 to 1.58)	(0.83 to 1.60)
Asian/PI	0.69	0.82	0.71	0.74	0.82	0.72	0.82	0.78
	(0.44 to 1.07)	(0.51 to 1.33)	(0.44 to 1.17)	(0.45 to 1.21)	(0.50 to 1.35)	(0.48 to 1.07)	(0.52 to 1.29)	(0.48 to 1.29)
NH, black, other, unknown	1.01	1.58	1.31	1.42	1.39	1.18	1.27	1.41
	(0.56 to 1.82)	(0.86 to 2.91)	(0.70 to 2.45)	(0.76 to 2.66)	(0.74 to 2.61)	(0.72 to 1.96)	(0.70 to 2.32)	(0.73 to 2.70)
Sex								
Female	1.00 (Referent)	1.00 (Referent)	1.00 (Referent)	1.00 (Referent)	1.00 (Referent)	1.00 (Referent)	1.00 (Referent)	1.00 (Referent)
Male	1.07	1.24	1.15	1.18	1.08	1.00	1.03	1.13
	(0.85 to 1.35)	(0.96 to 1.61)	(0.89 to 1.48)	(0.91 to 1.53)	(0.84 to 1.40)	(0.81 to 1.23)	(0.81 to 1.31)	(0.87 to 1.47)
Health insurance								
Private/military	1.00 (Referent)	1.00 (Referent)	1.00 (Referent)	1.00 (Referent)	1.00 (Referent)	1.00 (Referent)	1.00 (Referent)	1.00 (Referent)
Public/uninsured	1.43	1.57	1.49	1.49	1.65	1.40	1.44	1.40
	(1.12 to 1.83)	(1.20 to 2.07)	(1.14 to 1.95)	(1.13 to 1.97)	(1.25 to 2.17)	(1.12 to 1.76)	(1.12 to 1.86)	(1.06 to 1.86)
Unknown	1.02	1.06	0.76	1.18	1.03	1.05	0.88	0.94
	(0.58 to 1.80)	(0.56 to 2.02)	(0.38 to 1.53)	(0.63 to 2.24)	(0.54 to 1.95)	(0.63 to 1.76)	(0.47 to 1.65)	(0.48 to 1.85)
HCT								
No	1.00 (Referent)	1.00 (Referent)	1.00 (Referent)	1.00 (Referent)	1.00 (Referent)	1.00 (Referent)	1.00 (Referent)	1.00 (Referent)
Yes	2.62	2.99	3.01	3.32	2.73	2.15	2.54	3.57
	(2.04 to 3.36)	(2.28 to 3.91)	(2.28 to 3.97)	(2.52 to 4.35)	(2.08 to 3.59)	(1.70 to 2.72)	(1.96 to 3.27)	(2.70 to 4.73)
Year of diagnosis								
1995-2000	1.00 (Referent)	1.00 (Referent)	1.00 (Referent)	1.00 (Referent)	1.00 (Referent)	1.00 (Referent)	1.00 (Referent)	1.00 (Referent)
2001-2006	0.87	0.86	0.77	0.91	0.77	1.07	0.70	0.88
	(0.65 to 1.16)	(0.62 to 1.19)	(0.56 to 1.06)	(0.66 to 1.26)	(0.56 to 1.06)	(0.82 to 1.41)	(0.52 to 0.95)	(0.63 to 1.21)
2007-2012	1.23	1.22	0.98	1.20	1.06	1.48	1.02	1.06
	(0.90 to 1.68)	(0.86 to 1.73)	(0.70 to 1.38)	(0.84 to 1.70)	(0.75 to 1.50)	(1.11 to 1.98)	(0.75 to 1.40)	(0.74 to 1.51)
Neighborhood SES tertile								
Low	1.29	1.27	1.28	1.25	1.16	1.34	1.18	1.26
	(0.92 to 1.81)	(0.87 to 1.86)	(0.88 to 1.86)	(0.86 to 1.83)	(0.79 to 1.69)	(0.99 to 1.82)	(0.83 to 1.68)	(0.86 to 1.85)
Medium	1.12	1.22	1.24	1.23	1.13	1.08	1.15	1.28
	(0.83 to 1.53)	(0.86 to 1.73)	(0.88 to 1.74)	(0.87 to 1.74)	(0.79 to 1.60)	(0.82 to 1.43)	(0.83 to 1.59)	(0.90 to 1.81)
High	1.00 (Referent)	1.00 (Referent)	1.00 (Referent)	1.00 (Referent)	1.00 (Referent)	1.00 (Referent)	1.00 (Referent)	1.00 (Referent)
Cranial irradiation								
No	1.00 (Referent)	1.00 (Referent)	1.00 (Referent)	1.00 (Referent)	1.00 (Referent)	1.00 (Referent)	1.00 (Referent)	1.00 (Referent)
Yes	1.06	1.01	0.94	0.99	0.95	1.12	0.89	0.87
	(0.80 to 1.40)	(0.74 to 1.38)	(0.69 to 1.29)	(0.72 to 1.36)	(0.69 to 1.30)	(0.86 to 1.45)	(0.66 to 1.20)	(0.63 to 1.21)
Unknown	0.96	1.06	0.92	1.04	0.83	1.05	0.94	0.80
	(0.55 to 1.67)	(0.60 to 1.90)	(0.49 to 1.71)	(0.57 to 1.90)	(0.43 to 1.59)	(0.64 to 1.74)	(0.52 to 1.70)	(0.42 to 1.55)

* Each late effect column represents a separate multivariable model adjusted for all variables in the model and stratified by age group and location of care, which violated proportional hazards assumptions. ALL = acute lymphoblastic leukemia; AYA = adolescent and young adult; CI = confidence interval; HCT = hematopoietic cell transplant; HR = hazard ratio; NH = non-Hispanic; PI = Pacific Islander; SES = socioeconomic status.

**Table 4.** Multivariable-adjusted HR and associated 95% CI of medical conditions among 3-year AYA ALL survivors, California, 1995-2012, including only nontransplanted patients (n = 766 in each model)*

Variable	Cardiac diseases	Seizure/Stroke	Respiratory system diseases	Renal disease	Liver disease	Endocrine disease	Avascular events	Second cancers
	HR (95% CI)	HR (95% CI)	HR (95% CI)	HR (95% CI)	HR (95% CI)	HR (95% CI)	HR (95% CI)	HR (95% CI)
Race/ethnicity								
NH white	1.00 (Referent)	1.00 (Referent)	1.00 (Referent)	1.00 (Referent)	1.00 (Referent)	1.00 (Referent)	1.00 (Referent)	1.00 (Referent)
Hispanic	1.11	1.28	1.33	1.40	1.52	1.08	1.62	1.45
	(0.75 to 1.65)	(0.82 to 2.01)	(0.86 to 2.08)	(0.89 to 2.20)	(0.97 to 2.39)	(0.75 to 1.55)	(1.08 to 2.45)	(0.92 to 2.30)
Asian/PI	0.69	0.85	0.77	0.70	0.84	0.74	0.98	0.88
	(0.37 to 1.31)	(0.43 to 1.68)	(0.38 to 1.56)	(0.34 to 1.46)	(0.41 to 1.70)	(0.42 to 1.29)	(0.52 to 1.82)	(0.43 to 1.79)
NH, black, other, unknown	1.18	1.82	1.87	1.87	1.92	1.58	1.74	1.86
	(0.57 to 2.44)	(0.86 to 3.88)	(0.88 to 3.95)	(0.88 to 3.96)	(0.90 to 4.10)	(0.88 to 2.84)	(0.86 to 3.53)	(0.84 to 4.12)
Sex								
Female	1.00 (Referent)	1.00 (Referent)	1.00 (Referent)	1.00 (Referent)	1.00 (Referent)	1.00 (Referent)	1.00 (Referent)	1.00 (Referent)
Male	1.10	1.37	1.22	1.20	1.13	0.88	0.93	1.15
	(0.80 to 1.51)	(0.95 to 1.97)	(0.86 to 1.75)	(0.83 to 1.72)	(0.79 to 1.61)	(0.66 to 1.17)	(0.68 to 1.28)	(0.80 to 1.66)
Health insurance								
Private/military	1.00 (Referent)	1.00 (Referent)	1.00 (Referent)	1.00 (Referent)	1.00 (Referent)	1.00 (Referent)	1.00 (Referent)	1.00 (Referent)
Public/uninsured	1.73	1.63	1.76	1.72	1.85	1.59	1.69	1.59
	(1.24 to 2.42)	(1.13 to 2.36)	(1.22 to 2.54)	(1.18 to 2.50)	(1.28 to 2.67)	(1.17 to 2.15)	(1.21 to 2.36)	(1.09 to 2.33)
Unknown	0.84	0.60	0.49	0.88	0.65	0.95	0.66	0.58
	(0.39 to 1.80)	(0.23 to 1.54)	(0.17 to 1.37)	(0.37 to 2.10)	(0.26 to 1.66)	(0.49 to 1.84)	(0.28 to 1.55)	(0.22 to 1.52)
Year of diagnosis								
1995-2000	1.00 (Referent)	1.00 (Referent)	1.00 (Referent)	1.00 (Referent)	1.00 (Referent)	1.00 (Referent)	1.00 (Referent)	1.00 (Referent)
2001-2006	0.83	0.89	0.77	0.95	0.74	1.04	0.76	0.85
	(0.56 to 1.24)	(0.57 to 1.38)	(0.50 to 1.18)	(0.61 to 1.48)	(0.48 to 1.15)	(0.72 to 1.49)	(0.51 to 1.12)	(0.55 to 1.33)
2007-2012	1.15	1.22	0.95	1.13	1.07	1.47	0.99	1.01
	(0.75 to 1.76)	(0.77 to 1.94)	(0.60 to 1.49)	(0.70 to 1.81)	(0.68 to 1.68)	(0.99 to 2.17)	(0.66 to 1.49)	(0.63 to 1.62)
Neighborhood SES tertile								
Low	1.08	1.03	0.96	0.93	0.90	1.20	0.78	0.91
	(0.68 to 1.73)	(0.61 to 1.73)	(0.57 to 1.61)	(0.55 to 1.58)	(0.53 to 1.52)	(0.79 to 1.84)	(0.49 to 1.25)	(0.53 to 1.55)
Medium	0.93	0.98	0.95	0.97	1.00	1.01	0.85	0.94
	(0.61 to 1.42)	(0.61 to 1.56)	(0.60 to 1.52)	(0.61 to 1.56)	(0.62 to 1.61)	(0.69 to 1.49)	(0.55 to 1.29)	(0.58 to 1.51)
High	1.00 (Referent)	1.00 (Referent)	1.00 (Referent)	1.00 (Referent)	1.00 (Referent)	1.00 (Referent)	1.00 (Referent)	1.00 (Referent)
Frontline regimen								
Pediatric	1.00 (Referent)	1.00 (Referent)	1.00 (Referent)	1.00 (Referent)	1.00 (Referent)	1.00 (Referent)	1.00 (Referent)	1.00 (Referent)
Adult	1.00	0.68	0.82	0.73	0.87	0.79	0.90	0.83
	(0.60 to 1.68)	(0.37 to 1.23)	(0.46 to 1.46)	(0.41 to .33)	(0.49 to 1.55)	(0.49 to 1.27)	(0.54 to 1.48)	(0.46 to 1.52)
Unclear	0.73	0.58	0.63	0.72	0.69	0.81	0.78	0.69
	(0.35 to 1.53)	(0.25 to 1.34)	(0.27 to 1.44)	(0.32 to 1.60)	(0.30 to 1.59)	(0.44 to 1.48)	(0.40 to 1.53)	(0.30 to 1.60)
Cranial irradiation								
No	1.00 (Referent)	1.00 (Referent)	1.00 (Referent)	1.00 (Referent)	1.00 (Referent)	1.00 (Referent)	1.00 (Referent)	1.00 (Referent)
Yes	0.89	0.93	0.79	0.88	0.96	0.88	0.76	0.83
	(0.60 to 1.31)	(0.61 to 1.42)	(0.51 to 1.22)	(0.57 to 1.37)	(0.63 to 1.46)	(0.61 to 1.25)	(0.51 to 1.14)	(0.54 to 1.30)
Unknown	0.70	0.60	0.48	0.75	0.35	0.74	0.41	0.33
	(0.28 to 1.77)	(0.23 to 1.54)	(0.15 to 1.57)	(0.27 to 2.09)	(0.08 to 1.46)	(0.32 to 1.73)	(0.13 to 1.33)	(0.08 to 1.37)

* Each late effect column represents a separate multivariable model adjusted for all variables in the model and stratified by age group and location of care, which violated proportional hazards assumptions. ALL = acute lymphoblastic leukemia; AYA = adolescent and young adult; CI = confidence interval; HR = hazard ratio; NH = non-Hispanic; PI = Pacific Islander; SES = socioeconomic status.

**Table 5.** Multivariable-adjusted HR and associated 95% CI of medical conditions among 3-year AYA ALL survivors, California, 1995-2012, including only transplanted patients (n = 303 in each model)*

Variable	Cardiac diseases	Seizure/Stroke	Respiratory system diseases	Renal disease	Liver disease	Endocrine disease	Avascular events	Second cancers
	HR (95% CI)	HR (95% CI)	HR (95% CI)	HR (95% CI)	HR (95% CI)	HR (95% CI)	HR (95% CI)	HR (95% CI)
Race/ethnicity								
NH white	1.00 (Referent)	1.00 (Referent)	1.00 (Referent)	1.00 (Referent)	1.00 (Referent)	1.00 (Referent)	1.00 (Referent)	1.00 (Referent)
Hispanic	0.77	0.96	0.83	0.87	1.01	0.77	0.85	1.01
	(0.49 to 1.19)	(0.58 to 1.59)	(0.51 to 1.33)	(0.53 to 1.42)	(0.61 to 1.67)	(0.51 to 1.16)	(0.53 to 1.36)	(0.62 to 1.67)
Asian/PI	0.66	0.82	0.67	0.78	0.84	0.65	0.69	0.75
	(0.35 to 1.24)	(0.40 to 1.64)	(0.33 to 1.37)	(0.39 to 1.56)	(0.40 to 1.74)	(0.36 to 1.17)	(0.35 to 1.39)	(0.36 to 1.55)
NH, black, other, unknown	1.44	2.01	1.38	1.48	1.35	1.08	1.40	1.43
	(0.48 to 4.32)	(0.65 to 6.26)	(0.39 to 4.83)	(0.42 to 5.20)	(0.38 to 4.83)	(0.36 to 3.20)	(0.40 to 4.87)	(0.40 to 5.09)
Sex								
Female	1.00 (Referent)	1.00 (Referent)	1.00 (Referent)	1.00 (Referent)	1.00 (Referent)	1.00 (Referent)	1.00 (Referent)	1.00 (Referent)
Male	1.08	1.20	1.18	1.27	1.10	1.21	1.34	1.28
	(0.75 to 1.56)	(0.80 to 1.82)	(0.79 to 1.75)	(0.85 to 1.92)	(0.73 to 1.66)	(0.86 to 1.70)	(0.90 to 1.99)	(0.85 to 1.93)
Health insurance								
Private/military	1.00 (Referent)	1.00 (Referent)	1.00 (Referent)	1.00 (Referent)	1.00 (Referent)	1.00 (Referent)	1.00 (Referent)	1.00 (Referent)
Public/uninsured	1.00	1.23	1.02	1.02	1.17	1.11	0.95	0.96
	(0.67 to 1.48)	(0.79 to 1.91)	(0.67 to 1.55)	(0.66 to 1.57)	(0.75 to 1.83)	(0.77 to 1.59)	(0.62 to 1.45)	(0.62 to 1.49)
Unknown	1.97	3.54	2.28	2.26	2.68	1.92	2.13	2.76
	(0.79 to 4.93)	(1.37 to 9.19)	(0.87 to 5.97)	(0.86 to 5.94)	(1.09 to 6.60)	(0.81 to 4.60)	(0.82 to 5.58)	(1.04 to 7.32)
Year of diagnosis								
1995-2000	1.00 (Referent)	1.00 (Referent)	1.00 (Referent)	1.00 (Referent)	1.00 (Referent)	1.00 (Referent)	1.00 (Referent)	1.00 (Referent)
2001-2006	1.00	0.85	0.85	1.02	0.84	1.24	0.70	1.02
	(0.64 to 1.56)	(0.52 to 1.41)	(0.53 to 1.38)	(0.61 to 1.69)	(0.51 to 1.39)	(0.81 to 1.90)	(0.43 to 1.14)	(0.61 to 1.68)
2007-2012	1.39	1.24	1.13	1.44	1.14	1.54	1.13	1.35
	(0.86 to 2.26)	(0.71 to 2.15)	(0.67 to 1.92)	(0.83 to 2.49)	(0.65 to 1.98)	(0.98 to 2.43)	(0.68 to 1.89)	(0.78 to 2.36)
Neighborhood SES tertile								
Low	2.17	2.17	2.44	2.23	2.00	2.12	2.30	2.27
	(1.29 to 3.64)	( 1.18 to 3.99)	( 1.37 to 4.35)	(1.24 to 4.01)	( 1.10 to 3.63)	( 1.32 to 3.41)	( 1.30 to 4.05)	( 1.24 to 4.14)
Medium	1.83	2.28	2.51	2.13	1.77	1.59	2.24	2.31
	(1.13 to 2.98)	(1.28 to 4.04)	(1.46 to 4.33)	(1.22 to 3.72)	(1.00 to 3.14)	(1.01 to 2.49)	(1.30 to 3.85)	(1.31 to 4.06)
High	1.00 (Referent)	1.00 (Referent)	1.00 (Referent)	1.00 (Referent)	1.00 (Referent)	1.00 (Referent)	1.00 (Referent)	1.00 (Referent)
Frontline regimen								
Pediatric	1.00 (Referent)	1.00 (Referent)	1.00 (Referent)	1.00 (Referent)	1.00 (Referent)	1.00 (Referent)	1.00 (Referent)	1.00 (Referent)
Adult	0.47	0.63	0.51	0.50	0.56	0.71	0.70	0.74
	(0.24 to 0.92)	(0.28 to 1.42)	(0.24 to 1.06)	(0.23 to 1.06)	(0.24 to 1.30)	(0.39 to 1.31)	(0.33 to 1.47)	(0.34 to 1.65)
Unclear	0.64	0.81	0.81	0.54	0.55	0.73	0.94	0.77
	(0.27 to 1.51)	(0.29 to 2.27)	(0.33 to 1.99)	(0.19 to 1.49)	(0.19 to 1.63)	(0.33 to 1.62)	(0.36 to 2.45)	(0.27 to 2.18)
Cranial irradiation								
No	1.00 (Referent)	1.00 (Referent)	1.00 (Referent)	1.00 (Referent)	1.00 (Referent)	1.00 (Referent)	1.00 (Referent)	1.00 (Referent)
Yes	1.10	0.91	0.99	1.01	0.79	1.36	0.94	0.80
	(0.70 to 1.74)	(0.54 to 1.52)	(0.61 to 1.60)	(0.61 to 1.66)	(0.47 to 1.33)	(0.91 to 2.03)	(0.57 to 1.53)	(0.48 to 1.34)
Unknown	1.08	1.43	1.11	1.26	1.22	1.30	1.36	1.30
	(0.51 to 2.26)	(0.67 to 3.03)	(0.50 to 2.47)	(0.57 to 2.78)	(0.55 to 2.73)	(0.67 to 2.53)	(0.64 to 2.89)	(0.59 to 2.87)

* Each late effect column represents a separate multivariable model adjusted for all variables in the model and stratified by age group and location of care, which violated proportional hazards assumptions. ALL = acute lymphoblastic leukemia; AYA = adolescent and young adult; CI = confidence interval; HR = hazard ratio; NH = non-Hispanic; PI = Pacific Islander; SES = socioeconomic status.

All analyses were conducted using SAS version 9.4 software (SAS Institute Inc, Cary, NC). All statistical tests were 2-sided, and a *P* value of less than .05 was considered statistically significant.

## Results

### Study Population

The final study cohort included 1069 AYA patients (aged 15-39 years at diagnosis) with ALL diagnosed between 1995 and 2012 in California who survived at least 3 years following diagnosis and were successfully identified in both the CCR and the OSHPD databases (Figure 1). Baseline patient sociodemographics and ALL treatment characteristics are described in Table 1. The median age at diagnosis was 21 years; 61.9% were male, and 50.1% were Hispanic. Private health insurance coverage was reported in 58.6%, 35.0% had public insurance, and 2.3% were uninsured. Of the patients, 59.5% received an adult-type frontline ALL regimen, 19% received CRT, and 28.3% underwent HCT. The median time from diagnosis to last follow-up was 8.2 years (interquartile range = 4.9-13.8). At the time of last study follow-up, 218 (20.4%) patients had died; 87.6% of the deaths were attributed to ALL.

**Table 1.** Baseline characteristics among 3-year AYA ALL survivors, 1995-2012, California*

Characteristic	No. (%)
	(n = 1069)
Age at diagnosis, y	
15-19	469 (43.9)
20-29	343 (32.1)
30-39	257 (24.0)
Year of diagnosis	
1995-2000	303 (28.3)
2001-2006	342 (32.0)
2007-2012	424 (39.7)
Sex	
Female	407 (38.1)
Male	662 (61.9)
Race/ethnicity	
NH white	379 (35.5)
NH black	35 (3.3)
Hispanic	536 (50.1)
Asian/PI	110 (10.3)
Other/unknown	9 (0.8)
Neighborhood SES tertile	
1 lowest	416 (38.9)
2	359 (33.6)
3 highest	294 (27.5)
Health insurance	
Private/military	626 (58.6)
Public	374 (35.0)
Uninsured	24 (2.3)
Unknown	45 (4.2)
Frontline regimen	
Adult	636 (59.5)
Pediatric	353 (33.0)
Unclear	80 (7.5)
Location of care treatment facility at COG or NCI CC	
Always	578 (54.1)
Partial/none	469 (43.9)
Unknown	22 (2.1)
Cranial irradiation	
Yes	203 (19.0)
No	824 (77.1)
Unknown	42 (3.9)
Hematopoietic cell transplant	
Yes	303 (28.3)
No	766 (71.7)
Died before last study follow-up	218 (20.4)
Median (IQR) time to HCT, mo	7.5 (4.4-35.3)
Median (IQR) follow-up time, y	8.18 (4.9-13.8)

* ALL = acute lymphoblastic leukemia; AYA = adolescent and young adult; CC = cancer center; COG = Children’s Oncology Group; HCT = hematopoietic cell transplant; IQR = interquartile range; NCI = National Cancer Institute; NH = non-Hispanic; PI = Pacific Islander; SES = socioeconomic status.

### CMI Estimates of Late Effects

The estimated 5- and 10-year CMI of late effects are depicted in Table 2. The 10-year CMI of endocrine disease (28.7%, 95% CI = 25.8% to 31.6%) and cardiac disease (17.0%, 95% CI = 14.6% to 19.5%) were highest, followed by AVN (9.6%, 95% CI = 7.8% to 11.6%), liver disease (6.5%, 95% CI = 5.0% to 8.3%), respiratory disease (6.2%, 95% CI = 4.8% to 8.0%), seizure/stroke (4.3%, 95% CI = 3.1% to 5.8%), renal disease (3.1%, 95% CI = 2.1% to 4.4%), and SN (1.4%, 95% CI = 0.7% to 2.4%). Excluding nonmelanoma skin cancers, 19 SN were reported to have occurred (Supplementary Table 2, available online). All late effects increased over time, with a doubling of estimated SN between 5 and 10 years. There was no statistically significant difference in the CMI of late effects by frontline regimen type in the study cohort or in a sensitivity analysis excluding patients whose frontline regimen type was assigned by the study algorithm (Supplementary Table 3, available online). However, 10-year CMIs of late effects including SN were statistically significantly increased in AYAs who underwent HCT; unadjusted 10-year CMI of late effects by baseline demographic and clinical characteristics is shown in Supplementary Table 4 (available online).

**Table 2.** Cumulative incidence with 95% CI of late effects among 3-year AYA ALL survivors, 1995–2012, California*

Medical conditions	All patients	Pediatric frontline regimen	Adult frontline regimen
	% (95% CI)	% (95% CI)	% (95% CI)
	(n = 1069)	(n= 353)	(n = 636)
Second cancers			
5-year	0.4 (0.1 to 1.0)	0.3 (0.0 to 1.6)	0.5 (0.1 to 1.4)
10-year	1.4 (0.7 to 2.4)	0.3 (0.0 to 1.6)	2.1 (1.1 to 3.8)
Cardiac diseases			
5-year	10.5 (8.7 to 12.5)	10.9 (7.9 to 14.5)	10.6 (8.3 to 13.2)
10-year	17.0 (14.6 to 19.5)	15.8 (12.0 to 20.0)	17.5 (4.4 to 20.9)
Seizures/strokes			
5-year	2.4 (1.6 to 3.5)	2.6 (1.3 to 4.8)	2.5 (1.5 to 4.0)
10-year	4.3 (3.1 to 5.8)	3.7 (2.0 to 6.3)	4.5 (3.0 to 6.6)
Respiratory system diseases			
5-year	3.7 (2.7 to 5.0)	4.7 (2.8 to 7.3)	3.3 (2.1 to 5.0)
10-year	6.2 (4.8 to 8.0)	6.4 (4.1 to 9.4)	6.1 (4.3 to 8.4)
Renal disease			
5-year	1.9 (1.2 to 2.9)	2.0 (0.9 to 4.0)	2.1 (1.1 to 3.5)
10-year	3.1 (2.1 to 4.4)	3.4 (1.8 to 5.9)	3.3 (2.0 to 5.1)
Liver disease			
5-year	4.0 (2.9 to 5.3)	4.9 (3.0 to 7.6)	3.3 (2.1 to 4.9)
10-year	6.5 (5.0 to 8.3)	7.1 (4.6 to 10.3)	5.8 (4.0 to 8.1)
Endocrine disease			
5-year	19.3 (16.9 to 21.7)	17.5 (13.7 to 21.7)	20.7 (17.6 to 24.0)
10-year	28.7 (25.8 to 31.6)	26.0 (21.3 to 31.0)	30.1 (26.3 to 34.0)
Avascular events			
5-year	6.7 (5.2 to 8.3)	7.9 (5.3 to 11.0)	6.0 (4.3 to 8.1)
10-year	9.6 (7.8 to 11.6)	11.7 (8.4 to 15.5)	8.0 (5.9 to 10.4)

* ALL = acute lymphoblastic leukemia; AYA = adolescent and young adult; CI = confidence interval.

### Multivariable Analyses of Late Effects

Multivariable analysis including the entire study cohort (n = 1069) demonstrated that public or no health insurance (vs private/military) and receipt of HCT were statistically significantly and independently associated with every late effect considered in this study (Table 3). Statistically significant associations were also found between diagnosis time period and late effects, with AYAs diagnosed between 2007 and 2012 more likely to develop endocrine disease (HR = 1.48, 95% CI = 1.11 to 1.98) and AYAs diagnosed between 2001 and 2006 less likely to develop AVN (HR = 0.70, 95% CI = 0.52 to 0.95) compared with those diagnosed from 1995 to 2000.

Multivariable analysis among patients who were not transplanted (n = 766) similarly revealed public or no health insurance (vs private/military) to be statistically and independently associated with all late effects (Table 4). This analysis also demonstrated that AVN was statistically significantly increased in Hispanics (HR = 1.62, 95% CI = 1.08 to 2.45) relative to non-Hispanic whites. There were no statistically significant associations uncovered between frontline regimen type or use of CRT and any of the late effects categories studied.

Finally, a separate multivariable analysis among transplanted patients (n = 303) demonstrated that in this cohort, neighborhood SES was associated with a higher risk of all late effects considered (Table 5), with AYAs in the lowest 2 SES tertiles experiencing statistically significant increases in late effects relative to those in the highest SES tertile. Health insurance type and race and ethnicity were not statistically significantly associated with late effects in this cohort. Among transplanted patients, receipt of adult-type ALL frontline regimens was associated with a reduction in cardiac disease (HR = 0.47, 95% CI = 0.24 to 0.92). 

## Discussion

The spectacular success of childhood ALL has resulted not only from the careful development of effective therapeutic regimens but also from the methodical tracking and reporting of late effects following successful leukemia treatment. As the therapeutic management of AYA ALL evolves and more closely mirrors the pediatric approach, it is anticipated that a greater proportion of patients will become long-term survivors of AYA ALL. It is therefore increasingly important to more clearly ascertain the burden of late effects in this cancer survivor population. In this population-based analysis of survivors of AYA ALL across California, we demonstrate that the cumulative incidence of cardiac diseases (17%), endocrine diseases (28.7%), and AVN (9.6%) is estimated to be strikingly high at 10 years following diagnosis, and the incidence of SN was 1.4%. Further, we found that the development of late effects across all organ systems was statistically significantly and independently increased in uninsured or publicly insured survivors and AYAs who had undergone HCT.

In 2013, the Institute of Medicine (now the Health and Medicine Division of the National Academies of Sciences, Engineering, and Medicine) convened a workshop focused on addressing the needs of AYA cancer patients, which identified a critical gap in adequate research related to late effects following AYA cancers (16). Although the CCSS collects detailed long-term data on ALL patients diagnosed during childhood and adolescence (up to age 21 years) (17), understanding late effects after young adult ALL is important as well, as therapeutic exposures, lifestyle choices, and access to survivorship care may differ across the AYA ALL spectrum (5). To date, there has been a paucity of data related to the sequalae of ALL treated in young adulthood; therefore, survivorship guidelines for adult oncologists and internists have relied on extrapolating resources intended for survivors of childhood cancers. The data on survivors of AYA cancers in general do suggest that cancer survivors have a higher prevalence of asthma, chronic obstructive pulmonary disorder, stroke, and diabetes (18,19) and more than a twofold increased rate of cardiovascular disease (20) relative to those without a cancer history. We found the endocrine late effects appeared to increase over time, which may be a result of therapeutic interventions but may also reflect a growing burden of diabetes in the US AYA population over time.

In the current study, we aimed to describe late effects across organ systems and SN in patients who have survived at least 3 years following a diagnosis of AYA ALL and to evaluate associations between baseline sociodemographics and general treatment approaches with late effects. It is useful to examine our findings relative to late effects described in survivors of childhood ALL but challenging because of substantial differences in patient cohorts, follow-up time, and methodologies across studies. For example, the CCSS uses a survey-based assessment tool to track late effects, whereas we used a combination of cancer registry and hospitalization data. In a CCSS analysis with longer follow-up of survivors of childhood and adolescent ALL (younger than age 21 years at diagnosis) reported by Turcotte and colleagues (21), the 15-year cumulative incidence of SN among those diagnosed between 1990 and 1999 was 1.1 (95% CI = 0.8 to 1.4). Given that the incidence of SN appears to increase with longer follow-up, we anticipate that the rate of SN in our AYA population will be higher than what has been reported in survivors of childhood ALL. We found very high 10-year cumulative incidences of endocrine and cardiac late effects in our cohort. In a population-based cohort analysis of 1-year survivors of childhood cancers across Scandinavia, the study population was found to have a 4.8 times increase in the risk of having a hospital contact for an endocrine disorder, with the highest rates of endocrine late effects seen in survivors of childhood leukemia (22). The CCSS has performed detailed analyses of cardiac late effects with lower cumulative incidences than we report (23,24); however, we included hypertensive conditions in this category, whereas the CCSS generally has not.

It is well known that exposures to certain therapeutic agents and modalities influence the development of late effects following cancer therapy. Although children with ALL are routinely treated with multiagent pediatric ALL therapeutic backbones that incorporate substantial cumulative doses of steroids, vincristine, asparaginase, and intrathecal chemotherapy, AYAs may be treated with these pediatric-type ALL regimens or with adult-type ALL regimens, which generally include more anthracyclines and alkylating agents (6). It has been hypothesized that the distinctive agents used in the pediatric vs adult therapeutic ALL regimens may result in varying late effects; however, the influence of ALL regimen type on late effects in AYA ALL has not been previously described. In our dataset, in which approximately twice as many AYAs received an adult ALL regimen than a pediatric ALL regimen, we found no associations between regimen type and any of the late effects categories, with the exception of a reduction in cardiac late effects only among AYAs who received an adult frontline regimen and subsequent HCT. As we looked at late effects only among 3-year survivors, the lack of association between regimen and late effects may be influenced by differences in 3-year survival among AYAs treated with adult vs pediatric regimens. Although further follow-up time may be necessary to delineate subtle differences based on regimen, this result provides initial evidence that there are not substantial differences in the development of late effects among survivors based on the general type of frontline ALL regimen administered.

In contrast, we found that receipt of HCT was strongly associated with the development of late effects across all categories. Perhaps most marked was the increase in a 10-year cumulative incidence of SN with HCT (4% vs 0.3%). This is particularly striking because this dataset does not include nonmelanoma skin cancers, which have been previously reported as among the most frequent SN following HCT (25). The association between HCT, particularly among patients with chronic graft-vs-host disease, and late effects is well known (26,27), but few prior studies have compared late effects among a population of survivors who had undergone HCT and those who had not. Among patients who had undergone an HCT, lower SES was consistently associated with a higher risk of late effects across organ systems; however, among those who did not undergo an HCT, public or no health insurance, but not SES, was associated with a statistically significant increase in late effects. It may be that insurance status has less of an effect on late effects after HCT because these patients have all obtained insurance coverage for transplant and their subsequent care is in part managed by the transplant center. Health insurance and SES associations have been shown in other studies of late effects in a variety of cancer populations (28,29) and may reflect variations in lifestyle choices, access to care, and survivorship follow-up among survivors with differing health insurance coverage and economic resources. At a minimum, this finding suggests that survivors of AYA ALL who are uninsured or publicly insured or of lower SES are a high-risk population, and additional attention and investigation should focus on ensuring that these patients have high-quality survivorship care.

The use of population-based registries to approximate the incidence of late effects following cancer therapy has inherent strengths and limitations. Our analysis included linked statewide cancer registry and hospitalization data, which allowed us to evaluate important sociodemographic and cancer treatment details, as well as SN and late effects warranting hospitalization. However, we were only able to abstract the initial frontline regimen administered and were unable to detail the cumulative doses of specific therapeutic agents, including radiation therapy delivered outside the central nervous system. We also relied on diagnosis codes for late effects and were unable to capture diagnoses occurring during clinic visits, potentially underestimating the burden of late effects in our population. Similarly, we were unable to capture late effects that were diagnosed outside of the state of California. Additional studies are required to fully capture the burden of late effects that do not result in hospitalizations and those occurring on a national level. We were also unable to capture cytogenetic and molecular leukemic features at diagnosis, as well as lifestyle factors and survivorship plans that may influence late effects. Finally, the median follow-up time of 8.2 years is not long enough to assess the full burden of late effects in survivors of AYA ALL. It is well known that certain complications, including SN, may occur decades after the completion of therapy (4,30,31).

In conclusion, this study provides a large population-based description of late effects in survivors of AYA ALL. Given the nascent literature describing late effects, particularly in older AYAs treated in adult oncology settings, these data offer valuable insight into the burden of late effects in this vulnerable survivor population. Prospective population-based studies with longer follow-up would further inform the development of AYA-specific survivorship guidelines.

## Funding

This work was supported in part by Servier Pharmaceuticals. The collection of cancer incidence data used in this study was supported by the California Department of Public Health pursuant to California Health and Safety Code Section 103885; Centers for Disease Control and Prevention’s National Program of Cancer Registries, under cooperative agreement 5NU58DP006344; the National Cancer Institute’s Surveillance, Epidemiology, and End Results Program under contract HHSN261201800032I awarded to the University of California, San Francisco, contract HHSN261201800015I awarded to the University of Southern California, and contract HHSN261201800009I awarded to the Public Health Institute.

## Notes

**Role of the funder:** The funders had no role in the design of the study; the collection, analysis, and interpretation of the data; the writing of the manuscript; and the decision to submit the manuscript for publication.

**Disclaimers:** The ideas and opinions expressed herein are those of the author(s) and do not necessarily reflect the opinions of the State of California, Department of Public Health, the National Cancer Institute, and the Centers for Disease Control and Prevention or their contractors and subcontractors.

**Disclosures:** Lori Muffly reports consulting fees for Kite and Pfizer and research support from Servier and Adaptive. Frances Maguire, Qian Li, Vanessa Kennedy, and Theresa Keegan report no conflicts of interest.

## Supplementary Material

pkaa025_Supplementary_Data
