# Supplementary material for: Late Effects in Survivors of Adolescent and Young Adult Acute Lymphoblastic Leukemia
Source: JNCI Cancer Spectr. 2020 Apr 2;4(4):pkaa025. doi: 10.1093/jncics/pkaa025 (PMC7368465; doi:10.1093/jncics/pkaa025)
Supplement: pkaa025_Supplementary_Data [file pkaa025_supplementary_data.pdf]

Supplementary Table 1. ICD9 and ICD10 codes used for late effects

	ICD9	ICD10
Cardiac		
Hypertensive disease		
	401-405	I10., I16.0, I16.1, I16.9, I11.9, I11.0, I12.9, I12.0, I13.10, I13.0, I13.11, I13.2, I15.0, I15.8, I15.1, N26.2, I15.2, I15.9
Ischemic heart disease		
Diagnosis codes:		
	410-414	I21.09, I22.0, I21.01, I21.02, I21.19, I22.1, I21.11, I21.29, I22.8, I21.4, I22.2, I21.21, I21.3, I21.9, I21.A1, I21.A9, I22.9, I24.1, I20.0, I25.110, I25.700, I25.710, I25.720, I25.730, I25.750, I25.760, I25.790, I24.0, I24.8, I24.9, I25.2, I20.8, I20.1, I20.9, I25.111, I25.118, I25.119, I25.701, I25.708, I25.709, I25.711, I25.718, I25.719, I25.721, I25.728, I25.729, I25.731, I25.738, I25.739, I25.751, I25.758, I25.759, I25.761, I25.768, I25.769, I25.791, I25.798, I25.799, I25.10, I25.810, I25.811, I25.812, I25.3, I25.41, I25.42, I25.82, I25.83, I25.84, I25.5, I25.6, I25.89, I25.9
Procedure codes:		

V45.81, V45.82, 00.66, 35.41, 35.73, 35.84, 37.49, 37.67, 37.91, 37.99, 36.03, 36.04, 36.06, 36.07, 36.09, 36.10, 36.11, 36.12, 36.13, 36.14, 36.15, 36.16, 36.17, 36.19, 36.2, 36.31, 36.32, 36.33, 36.34, 36.39, 36.91, 36.99	Z95.1, Z95.5, Z98.61, 0270346, 027034Z, 0270356, 027035Z, 0270366, 027036Z, 0270376, 027037Z, 02703D6, 02703DZ, 02703E6, 02703EZ, 02703F6, 02703FZ, 02703G6, 02703GZ, 02703T6, 02703TZ, 02703Z6, 02703ZZ, 0270446, 027044Z, 0270456, 027045Z, 0270466, 027046Z, 0270476, 027047Z, 02704D6, 02704DZ, 02704E6, 02704EZ, 02704F6, 02704FZ, 02704G6, 02704GZ, 02704T6, 02704TZ, 02704Z6, 02704ZZ, 0271346, 027134Z, 0271356, 027135Z, 0271366, 027136Z, 0271376, 027137Z, 02713D6, 02713DZ, 02713E6, 02713EZ, 02713F6, 02713FZ, 02713G6, 02713GZ, 02713T6, 02713TZ, 02713Z6, 02713ZZ, 0271446, 027144Z, 0271456, 027145Z, 0271466, 027146Z, 0271476, 027147Z, 02714D6, 02714DZ, 02714E6, 02714EZ, 02714F6, 02714FZ, 02714G6, 02714GZ, 02714T6, 02714TZ, 02714Z6, 02714ZZ, 0272346, 027234Z, 0272356, 027235Z, 0272366, 027236Z, 0272376, 027237Z, 02723D6, 02723DZ, 02723E6, 02723EZ, 02723F6, 02723FZ, 02723G6, 02723GZ, 02723T6, 02723TZ, 02723Z6, 02723ZZ, 0272446, 027244Z, 0272456, 027245Z, 0272466, 027246Z, 0272476, 027247Z, 02724D6, 02724DZ, 02724E6, 02724EZ, 02724F6, 02724FZ, 02724G6, 02724GZ, 02724T6, 02724TZ, 02724Z6, 02724ZZ, 0273346, 027334Z, 0273356, 027335Z, 0273366, 027336Z, 0273376, 027337Z, 02733D6, 02733DZ, 02733E6, 02733EZ, 02733F6, 02733FZ, 02733G6, 02733GZ, 02733T6, 02733TZ, 02733Z6, 02733ZZ, 0273446, 027344Z, 0273456, 027345Z, 0273466, 027346Z, 0273476, 027347Z, 02734D6, 02734DZ, 02734E6, 02734EZ, 02734F6, 02734FZ, 02734G6, 02734GZ, 02734T6, 02734TZ, 02734Z6, 02734ZZ, 0270046, 027004Z, 0270056, 027005Z, 0270066, 027006Z, 0270076, 027007Z, 02700D6, 02700DZ, 02700E6, 02700EZ, 02700F6, 02700FZ, 02700G6, 02700GZ, 02700T6, 02700TZ, 02700Z6, 02700ZZ, 0271046, 027104Z, 0271056, 027105Z, 0271066, 027106Z, 0271076, 027107Z, 02710D6, 02710DZ, 02710E6, 02710EZ, 02710F6, 02710FZ, 02710G6, 02710GZ, 02710T6, 02710TZ, 02710Z6, 02710ZZ, 0272046, 027204Z, 0272056, 027205Z, 0272066, 027206Z, 0272076, 027207Z, 02720D6, 02720DZ, 02720E6, 02720EZ, 02720F6, 02720FZ, 02720G6, 02720GZ, 02720T6, 02720TZ, 02720Z6, 02720ZZ, 0273046, 027304Z, 0273056, 027305Z, 0273066, 027306Z, 0273076, 027307Z, 02730D6, 02730DZ, 02730E6, 02730EZ, 02730F6, 02730FZ, 02730G6, 02730GZ, 02730T6, 02730TZ, 02730Z6, 02730ZZ, 02C00Z6, 02C00ZZ, 02C10Z6, 02C10ZZ, 02C20Z6, 02C20ZZ, 02C30Z6, 02C30ZZ, 3E07017, 3E070PZ, 3E07317, 3E073PZ, 02C03Z6, 02C03ZZ, 02C04Z6, 02C04ZZ, 02C13Z6, 02C13ZZ, 02C14Z6, 02C14ZZ, 02C23Z6, 02C23ZZ, 02C24Z6, 02C24ZZ, 02C33Z6, 02C33ZZ, 02C34Z6, 02C34ZZ, 0210093, 02100A3, 02100J3, 02100K3, 02100Z3, 0210493, 02104A3, 02104J3, 02104K3, 02104Z3, 021008W, 021009W, 02100AW, 02100JW, 02100KW, 021048W, 021049W, 02104AW, 02104JW, 02104KW, 021108W, 021109W, 02110AW, 02110JW, 02110KW, 021148W, 021149W, 02114AW, 02114JW, 02114KW, 021208W, 021209W, 02120AW, 02120JW, 02120KW, 021248W, 021249W, 02124AW, 02124JW, 02124KW, 021308W, 021309W, 02130AW, 02130JW, 02130KW, 021348W, 021349W, 02134AW, 02134JW, 02134KW, 0210088, 0210089, 021008C, 0210098, 0210099, 021009C, 02100A8, 02100A9, 02100AC, 02100J8, 02100J9, 02100JC, 02100K8, 02100K9, 02100KC, 02100Z8, 02100Z9, 02100ZC, 0210488, 0210489, 021048C, 0210498, 0210499, 021049C, 02104A8, 02104A9, 02104AC, 02104J8, 02104J9, 02104JC, 02104K8, 02104K9, 02104KC, 02104Z8, 02104Z9, 02104ZC, 0211088, 0211089, 021108C, 0211098, 0211099, 021109C, 02110A8, 02110A9, 02110AC, 02110J8, 02110J9, 02110JC, 02110K8, 02110K9, 02110KC, 02110Z8, 02110Z9, 02110ZC, 0211488, 0211489, 021148C, 0211498, 0211499, 021149C, 02114A8, 02114A9, 02114AC, 02114J8, 02114J9, 02114JC, 02114K8, 02114K9, 02114KC, 02114Z8, 02114Z9, 02114ZC, 0212088, 0212089, 021208C, 0212098, 0212099, 021209C, 02120A8, 02120A9, 02120AC, 02120J8, 02120J9, 02120JC, 02120K8, 02120K9, 02120KC, 02120Z8, 02120Z9, 02120ZC, 0212488, 0212489, 021248C, 0212498, 0212499, 021249C, 02124A8, 02124A9, 02124AC, 02124J8, 02124J9, 02124JC, 02124K8, 02124K9, 02124KC, 02124Z8, 02124Z9, 02124ZC, 0213088, 0213089, 021308C, 0213098, 0213099, 021309C, 02130A8, 02130A9, 02130AC, 02130J8, 02130J9, 02130JC, 02130K8, 02130K9, 02130KC, 02130Z8, 02130Z9, 02130ZC, 0213488, 0213489, 021348C, 0213498, 0213499, 021349C, 02134A8, 02134A9, 02134AC, 02134J8, 02134J9, 02134JC, 02134K8, 02134K9, 02134KC, 02134Z8, 02134Z9, 02134ZC, 021008F, 021009F, 02100AF, 02100JF, 02100KF, 02100ZF, 021048F, 021049F, 02104AF, 02104JF, 02104KF, 02104ZF, 021108F, 021109F, 02110AF, 02110JF, 02110KF, 02110ZF, 021148F, 021149F, 02114AF, 02114JF, 02114KF, 02114ZF, 021208F, 021209F, 02120AF, 02120JF, 02120KF, 02120ZF, 021248F, 021249F, 02124AF, 02124JF, 02124KF, 02124ZF, 021308F, 021309F, 02130AF, 02130JF, 02130KF, 02130ZF,

021348F, 021349F, 02134AF, 02134JF, 02134KF, 02134ZF, 0210083, 0210483, 0211083, 0211093, 02110A3, 02110J3, 02110K3, 02110Z3, 0211483, 0211493, 02114A3, 02114J3, 02114K3, 02114Z3, 0212083, 0212093, 02120A3, 02120J3, 02120K3, 02120Z3, 0212483, 0212493, 02124A3, 02124J3, 02124K3, 02124Z3, 0213083, 0213093, 02130A3, 02130J3, 02130K3, 02130Z3, 0213483, 0213493, 02134A3, 02134J3, 02134K3, 02134Z3, 021K0Z8, 021K0Z9, 021K0ZC, 021K0ZF, 021K0ZW, 021K4Z8, 021K4Z9, 021K4ZC, 021K4ZF, 021K4ZW, 021L08P, 021L08Q, 021L08R, 021L09P, 021L09Q, 021L09R, 021L0AP, 021L0AQ, 021L0AR, 021L0JP, 021L0JQ, 021L0JR, 021L0KP, 021L0KQ, 021L0KR, 021L0Z8, 021L0Z9, 021L0ZC, 021L0ZF, 021L0ZP, 021L0ZQ, 021L0ZR, 021L48P, 021L48Q, 021L48R, 021L49P, 021L49Q, 021L49R, 021L4AP, 021L4AQ, 021L4AR, 021L4JP, 021L4JQ, 021L4JR, 021L4KP, 021L4KQ, 021L4KR, 021L4Z8, 021L4Z9, 021L4ZC, 021L4ZF, 021L4ZP, 021L4ZQ, 021L4ZR, 021K0Z5, 021L0Z5, 021K4Z5, 021L4Z5, 02QA4ZZ, 02QB4ZZ, 02QC4ZZ, 02QA3ZZ, 02QB3ZZ, 02QC3ZZ, 0210344, 02103D4, 0210444, 02104D4, 0211344, 02113D4, 0211444, 02114D4, 0212344, 02123D4, 0212444, 02124D4, 0213344, 02133D4, 0213444, 02134D4, 02QA0ZZ, 02QB0ZZ, 02QC0ZZ, 02Q00ZZ, 02Q03ZZ, 02Q04ZZ, 02Q10ZZ, 02Q13ZZ, 02Q14ZZ, 02Q20ZZ, 02Q23ZZ, 02Q24ZZ, 02Q30ZZ, 02Q33ZZ, 02Q34ZZ, 02Q40ZZ, 02Q43ZZ, 02Q44ZZ, 02540ZZ, 02543ZZ, 02544ZZ, 02B40ZZ, 02B43ZZ, 02B44ZZ, 02C40ZZ, 02C43ZZ, 02C44ZZ, 02H402Z, 02H403Z, 02H40DZ, 02H40YZ, 02H43DZ, 02H43YZ, 02H442Z, 02H443Z, 02H44DZ, 02H44YZ, 02N00ZZ, 02N03ZZ, 02N04ZZ, 02N10ZZ, 02N13ZZ, 02N14ZZ, 02N20ZZ, 02N23ZZ, 02N24ZZ, 02N30ZZ, 02N33ZZ, 02N34ZZ, 02S00ZZ, 02S10ZZ

Other heart diseases (Including Cardiomyopathy HF)

Diagnosis codes:

420-429

I32., I30.1, I30.9, I30.0, I30.8, I33.0, I39., I33.9, I41., I40.9, I40.0, I40.1, I40.8, I31.2, I31.0, I31.1, I31.4, I31.8, I31.3, I31.9, I34.0, I34.1, I34.2, I34.8, I34.9, I35.0, I35.1, I35.2, I35.8, I35.9, I36.0, I36.1, I36.2, I36.8, I36.9, I37.0, I37.1, I37.2, I37.8, I37.9, I38., I42.3, I42.1, I42.2, I42.8, I42.4, I42.0, I42.5, I42.9, I42.6, I43., I42.7, I44.2, I44.30, I44.0, I44.1, I44.4, I44.5, I44.60, I44.69, I44.7, I45.0, I45.10, I45.19, I44.39, I45.4, I45.2, I45.3, I45.5, I45.6, I45.81, I45.89, I45.9, I47.1, I49.2, I47.0, I47.2, I47.9, I48.0, I48.1, I48.2, I48.91, I48.3, I48.4, I48.92, I49.01, I49.02, I46.2, I46.8, I46.9, I49.40, I49.1, I49.3, I49.49, I49.5, I49.8, I49.9, I50.20, I50.21, I50.22, I50.23, I50.30, I50.31, I50.32, I50.33, I50.40, I50.41, I50.42, I50.43, I50.814, I50.9, I50.1, I50.810, I50.811, I50.812, I50.813, I50.82, I50.83, I50.84, I50.89, I51.4, I51.5, I25.10, I51.7, I97.0, I97.110, I97.111, I97.120, I97.121, I97.130, I97.131, I97.190, I97.191, I23.4, I51.1, I23.5, I51.2, I23.1, I23.2, I51.0, I23.0, I23.3, I23.6, I23.7, I23.8, I51.89, I51.81, I51.3, I51.9, I52.

Procedure codes:

	37.51, 33.6, V42.1	02YA0Z0, 02YA0Z1, 02YA0Z2, Z48.21, Z48.280, Z94.1, Z94.3
Neurologic disease: seizure and stroke		
Seizure	345	G40.A01, G40.A09, G40.A11, G40.A19, G40.309, G40.401, G40.409, G40.311, G40.319, G40.411, G40.419, G40.301, G40.201, G40.209, G40.211, G40.219, G40.001, G40.009, G40.101, G40.109, G40.011, G40.019, G40.111, G40.119, G40.821, G40.822, G40.823, G40.824, G40.501, G40.509, G40.801, G40.802, G40.811, G40.812, G40.89, G40.B01, G40.B09, G40.803, G40.804, G40.813, G40.814, G40.B11, G40.B19, G40.901, G40.909, G40.911, G40.919
Stroke		
Diagnosis codes:	430-435	I60.00, I60.01, I60.02, I60.10, I60.11, I60.12, I60.2, I60.30, I60.31, I60.32, I60.4, I60.50, I60.51, I60.52, I60.6, I60.7, I60.8, I60.9, I61.0, I61.1, I61.2, I61.3, I61.4, I61.5, I61.6, I61.8, I61.9, I62.1, I62.00, I62.01, I62.02, I62.03, I62.9, I65.1, I63.02, I63.12, I63.22, I65.21, I65.22, I65.23, I65.29, I63.031, I63.032, I63.033, I63.039, I63.131, I63.132, I63.133, I63.139, I63.231, I63.232, I63.233, I63.239, I65.01, I65.02, I65.03, I65.09, I63.011, I63.012, I63.013, I63.019, I63.111, I63.112, I63.113, I63.119, I63.211, I63.212, I63.213, I63.219, I65.8, I63.59, I63.09, I63.19, I65.9, I63.00, I63.10, I63.20, I63.29, I66.01, I66.02, I66.03, I66.09, I66.11, I66.12, I66.13, I66.19, I66.21, I66.22, I66.23, I66.29, I66.3, I63.30, I63.311, I63.312, I63.313, I63.319, I63.321, I63.322, I63.323, I63.329, I63.331, I63.332, I63.333, I63.339, I63.341, I63.342, I63.343, I63.349, I63.39, I63.6, I66.9, I63.40, I63.411, I63.412, I63.413, I63.419, I63.421, I63.422, I63.423, I63.429, I63.431, I63.432, I63.433, I63.439, I63.441, I63.442, I63.443, I63.449, I63.49, I66.8, I63.50, I63.511, I63.512, I63.513, I63.519, I63.521, I63.522, I63.523, I63.529, I63.531, I63.532, I63.533, I63.539, I63.541, I63.542, I63.543, I63.549, I63.8, I63.9, G45.0, G45.8, G45.1, G45.2, G46.0, G46.1, G46.2, G45.9, I67.841, I67.848
Procedure codes:	00.61-00.65, 39.74, 39.5, 39.90	037H346, 037H34Z, 037H356, 037H35Z, 037H366, 037H36Z, 037H376, 037H37Z, 037H3D6, 037H3DZ, 037H3E6, 037H3EZ, 037H3F6, 037H3FZ, 037H3G6, 037H3GZ, 037H3Z6, 037H3ZZ, 037H446, 037H44Z, 037H456, 037H45Z, 037H466, 037H46Z, 037H476, 037H47Z, 037H4D6, 037H4DZ, 037H4E6, 037H4EZ, 037H4F6, 037H4FZ, 037H4G6, 037H4GZ, 037H4Z6, 037H4ZZ, 037J346, 037J34Z, 037J356, 037J35Z, 037J366, 037J36Z, 037J376, 037J37Z, 037J3D6, 037J3DZ, 037J3E6, 037J3EZ, 037J3F6, 037J3FZ, 037J3G6, 037J3GZ, 037J3Z6, 037J3ZZ, 037J446, 037J44Z, 037J456, 037J45Z, 037J466, 037J46Z, 037J476, 037J47Z, 037J4D6, 037J4DZ, 037J4E6, 037J4EZ, 037J4F6, 037J4FZ, 037J4G6, 037J4GZ, 037J4Z6, 037J4ZZ, 037K346, 037K34Z, 037K356, 037K35Z, 037K366, 037K36Z, 037K376, 037K37Z, 037K3D6, 037K3DZ, 037K3E6, 037K3EZ, 037K3F6, 037K3FZ, 037K3G6, 037K3GZ, 037K3Z6, 037K3ZZ, 037K446, 037K44Z, 037K456, 037K45Z, 037K466, 037K46Z, 037K476, 037K47Z, 037K4D6, 037K4DZ, 037K4E6, 037K4EZ, 037K4F6, 037K4FZ, 037K4G6, 037K4GZ, 037K4Z6, 037K4ZZ, 037L346, 037L34Z, 037L356, 037L35Z, 037L366, 037L36Z, 037L376, 037L37Z, 037L3D6, 037L3DZ, 037L3E6, 037L3EZ, 037L3F6, 037L3FZ, 037L3G6, 037L3GZ, 037L3Z6, 037L3ZZ, 037L446, 037L44Z, 037L456, 037L45Z, 037L466, 037L46Z, 037L476, 037L47Z, 037L4D6, 037L4DZ, 037L4E6, 037L4EZ, 037L4F6, 037L4FZ, 037L4G6, 037L4GZ, 037L4Z6, 037L4ZZ, 037M346, 037M34Z, 037M356, 037M35Z, 037M366, 037M36Z, 037M376, 037M37Z, 037M3D6, 037M3DZ, 037M3E6, 037M3EZ, 037M3F6, 037M3FZ, 037M3G6, 037M3GZ, 037M3Z6, 037M3ZZ, 037M446, 037M44Z, 037M456, 037M45Z, 037M466, 037M46Z, 037M476, 037M47Z, 037M4D6, 037M4DZ, 037M4E6, 037M4EZ, 037M4F6, 037M4FZ, 037M4G6, 037M4GZ, 037M4Z6, 037M4ZZ, 037N346, 037N34Z, 037N356, 037N35Z, 037N366, 037N36Z, 037N376, 037N37Z, 037N3D6, 037N3DZ, 037N3E6, 037N3EZ, 037N3F6, 037N3FZ, 037N3G6, 037N3GZ, 037N3Z6, 037N3ZZ, 037N446, 037N44Z, 037N456, 037N45Z, 037N466, 037N46Z, 037N476, 037N47Z,

		037N4D6, 037N4DZ, 037N4E6, 037N4EZ, 037N4F6, 037N4FZ, 037N4G6, 037N4GZ, 037N4Z6, 037N4ZZ, 037P346, 037P34Z, 037P356, 037P35Z, 037P366, 037P36Z, 037P376, 037P37Z, 037P3D6, 037P3DZ, 037P3E6, 037P3EZ, 037P3F6, 037P3FZ, 037P3G6, 037P3GZ, 037P3Z6, 037P3ZZ, 037P446, 037P44Z, 037P456, 037P45Z, 037P466, 037P46Z, 037P476, 037P47Z, 037P4D6, 037P4DZ, 037P4E6, 037P4EZ, 037P4F6, 037P4FZ, 037P4G6, 037P4GZ, 037P4Z6, 037P4ZZ, 037Q346, 037Q34Z, 037Q356, 037Q35Z, 037Q366, 037Q36Z, 037Q376, 037Q37Z, 037Q3D6, 037Q3DZ, 037Q3E6, 037Q3EZ, 037Q3F6, 037Q3FZ, 037Q3G6, 037Q3GZ, 037Q3Z6, 037Q3ZZ, 037Q446, 037Q44Z, 037Q456, 037Q45Z, 037Q466, 037Q46Z, 037Q476, 037Q47Z, 037Q4D6, 037Q4DZ, 037Q4E6, 037Q4EZ, 037Q4F6, 037Q4FZ, 037Q4G6, 037Q4GZ, 037Q4Z6, 037Q4ZZ, 03CH3Z6, 03CH3ZZ, 03CJ3Z6, 03CJ3ZZ, 03CK3Z6, 03CK3ZZ, 03CL3Z6, 03CL3ZZ, 03CM3Z6, 03CM3ZZ, 03CN3Z6, 03CN3ZZ, 03CP3Z6, 03CP3ZZ, 03CQ3Z6, 03CQ3ZZ, 057M3DZ, 057M4DZ, 057N3DZ, 057N4DZ, 057P3DZ, 057P4DZ, 057Q3DZ, 057Q4DZ, 057R3DZ, 057R4DZ, 057S3DZ, 057S4DZ, 057T3DZ, 057T4DZ, 037G346, 037G34Z, 037G356, 037G35Z, 037G366, 037G36Z, 037G376, 037G37Z, 037G3D6, 037G3DZ, 037G3E6, 037G3EZ, 037G3F6, 037G3FZ, 037G3G6, 037G3GZ, 037G3Z6, 037G3ZZ, 037G446, 037G44Z, 037G456, 037G45Z, 037G466, 037G46Z, 037G476, 037G47Z, 037G4D6, 037G4DZ, 037G4E6, 037G4EZ, 037G4F6, 037G4FZ, 037G4G6, 037G4GZ, 037G4Z6, 037G4ZZ, 057L3DZ, 057L4DZ, 037R3D6, 037R3DZ, 037R3E6, 037R3EZ, 037R3F6, 037R3FZ, 037R3G6, 037R3GZ, 037R4D6, 037R4DZ, 037R4E6, 037R4EZ, 037R4F6, 037R4FZ, 037R4G6, 037R4GZ, 037S3D6, 037S3DZ, 037S3E6, 037S3EZ, 037S3F6, 037S3FZ, 037S3G6, 037S3GZ, 037S4D6, 037S4DZ, 037S4E6, 037S4EZ, 037S4F6, 037S4FZ, 037S4G6, 037S4GZ, 037T3D6, 037T3DZ, 037T3E6, 037T3EZ, 037T3F6, 037T3FZ, 037T3G6, 037T3GZ, 037T4D6, 037T4DZ, 037T4E6, 037T4EZ, 037T4F6, 037T4FZ, 037T4G6, 037T4GZ, 03CG3Z6, 03CG3ZZ, 03CG4Z6, 03CG4ZZ, 03CH4Z6, 03CH4ZZ, 03CJ4Z6, 03CJ4ZZ, 03CK4Z6, 03CK4ZZ, 03CL4Z6, 03CL4ZZ, 03CM4Z6, 03CM4ZZ, 03CN4Z6, 03CN4ZZ, 03CP4Z6, 03CP4ZZ, 03CQ4Z6, 03CQ4ZZ, 03CR3Z6, 03CR3ZZ, 03CR4Z6, 03CR4ZZ, 03CS3Z6, 03CS3ZZ, 03CS4Z6, 03CS4ZZ, 03CT3Z6, 03CT3ZZ, 03CT4Z6, 03CT4ZZ, 03CU3Z6, 03CU3ZZ, 03CU4Z6, 03CU4ZZ, 03CV3Z6, 03CV3ZZ, 03CV4Z6, 03CV4ZZ, 05CL3ZZ
Respiratory/Pulmonary		
Asthma		
	493	J45.20, J45.30, J45.40, J45.50, J45.22, J45.32, J45.42, J45.52, J45.21, J45.31, J45.41, J45.51, J45.990, J45.991, J45.909, J45.998, J45.902, J45.901
Chronic obstructive pulmonary disease and allied conditions		
	490-496	J40., J41.0, J41.1, J44.9, J44.1, J44.0, J41.8, J42., J43.9, J43.0, J43.1, J43.2, J43.8, J45.20, J45.30, J45.40, J45.50, J45.22, J45.32, J45.42, J45.52, J45.21, J45.31, J45.41, J45.51, J45.990, J45.991, J45.909, J45.998, J45.902, J45.901, J47.9, J47.0, J47.1, J67.0, J67.1, J67.2, J67.3, J67.4, J67.5, J67.6, J67.7, J67.8, J67.9
Pulmonary fibrosis		
	515	J84.10, J84.17, J84.89
Pneumonopathy		
	516	J84.01, J84.03, J84.02, J84.111, J84.112, J84.113, J84.114, J84.115, J84.2, J84.116, J84.117, J84.81, J84.82, J84.841, J84.842, J84.83, J84.843, J84.848, J84.09, J84.9
Renal		
Hypertensive		
	403, 404	I12.9, I12.0, I13.10, I13.0, I13.11, I13.2
Chronic kidney disease		
	585	N18.1, N18.2, N18.3, N18.4, N18.5, N18.6, N18.9
Kidney transplant		

	V42.0, 996.81	T86.10, T86.11, T86.12, T86.13, T86.19, Z48.22, Z94.0
hemodialysis		
	792.5, 458.21, V56.31, V45.11	I95.3, R88.0, Z99.2, Z49.31
Liver		
Chronic liver disease and cirrhosis		
	571.4, 571.5, 571.6, 571.8, 571.9, 572.2, 572.3, 572.4, 572.8, 570, 573.8, 573.9	K73.9, K73.0, K75.4, K73.1, K73.2, K73.8, K74.0, K74.60, K74.69, K74.3, K74.4, K74.5, K75.81, K76.0, K76.89, K74.1, K74.2, K76.9, K70.41, K71.11, K72.01, K72.11, K72.90, K72.91, K76.6, K76.7, K72.10
Chronic pancreatitis		
	577.1, 577.8	K86.0, K86.1, K86.81
Liver transplant		
	V42.7, 996.82, V58.44	T86.40, T86.41, T86.42, T86.43, T86.49, Z48.23, Z94.4
Hypothyroidism		
	244	E89.0, E03.2, E01.8, E02., E03.3, E03.8, E03.9
Diseases of the thyroid gland		
	240-243, 245, 246	E04.0, E01.0, E01.2, E04.9, E04.1, E01.1, E04.2, E04.8, E05.00, E05.01, E05.10, E05.11, E05.20, E05.21, E05.30, E05.31, E05.40, E05.80, E05.41, E05.81, E05.90, E05.91, E00.0, E00.1, E00.2, E00.9, E03.0, E03.1, E06.0, E06.1, E06.3, E06.5, E06.4, E06.2, E06.9, E07.0, E07.1, E07.89, E03.4, E35., E07.9
Diabetes mellitus		

	249, 250 , 349.89 , 353.5 , 355.9 , 357.2 , 361.00 , 361.81 , 362.01-362.07 , 366.41 , 443.81 , 523.8 , 528.9 , 536.3 , 581.81 , 583.81 , 709.8 , 713.5 , 716.8 , 785.4	E08.9, E09.9, E13.9, E08.65, E09.65, E08.10, E09.10, E13.10, E08.00, E08.01, E09.00, E09.01, E13.00, E13.01, E08.11, E08.641, E09.11, E09.641, E13.11, E13.641, E08.21, E08.22, E08.29, E09.21, E09.22, E09.29, E13.21, E13.22, E13.29, E08.311, E08.319, E08.3211, E08.3212, E08.3213, E08.3219, E08.3291, E08.3292, E08.3293, E08.3299, E08.3311, E08.3312, E08.3313, E08.3319, E08.3391, E08.3392, E08.3393, E08.3399, E08.3411, E08.3412, E08.3413, E08.3419, E08.3491, E08.3492, E08.3493, E08.3499, E08.3511, E08.3512, E08.3513, E08.3519, E08.3521, E08.3522, E08.3523, E08.3529, E08.3531, E08.3532, E08.3533, E08.3539, E08.3541, E08.3542, E08.3543, E08.3549, E08.3551, E08.3552, E08.3553, E08.3559, E08.3591, E08.3592, E08.3593, E08.3599, E08.36, E08.37X1, E08.37X2, E08.37X3, E08.37X9, E08.39, E09.311, E09.319, E09.3211, E09.3212, E09.3213, E09.3219, E09.3291, E09.3292, E09.3293, E09.3299, E09.3311, E09.3312, E09.3313, E09.3319, E09.3391, E09.3392, E09.3393, E09.3399, E09.3411, E09.3412, E09.3413, E09.3419, E09.3491, E09.3492, E09.3493, E09.3499, E09.3511, E09.3512, E09.3513, E09.3519, E09.3521, E09.3522, E09.3523, E09.3529, E09.3531, E09.3532, E09.3533, E09.3539, E09.3541, E09.3542, E09.3543, E09.3549, E09.3551, E09.3552, E09.3553, E09.3559, E09.3591, E09.3592, E09.3593, E09.3599, E09.36, E09.37X1, E09.37X2, E09.37X3, E09.37X9, E09.39, E13.311, E13.319, E13.3211, E13.3212, E13.3213, E13.3219, E13.3291, E13.3292, E13.3293, E13.3299, E13.3311, E13.3312, E13.3313, E13.3319, E13.3391, E13.3392, E13.3393, E13.3399, E13.3411, E13.3412, E13.3413, E13.3419, E13.3491, E13.3492, E13.3493, E13.3499, E13.3511, E13.3512, E13.3513, E13.3519, E13.3521, E13.3522, E13.3523, E13.3529, E13.3531, E13.3532, E13.3533, E13.3539, E13.3541, E13.3542, E13.3543, E13.3549, E13.3551, E13.3552, E13.3553, E13.3559, E13.3591, E13.3592, E13.3593, E13.3599, E13.36, E13.37X1, E13.37X2, E13.37X3, E13.37X9, E13.39, E08.40, E08.41, E08.42, E08.43, E08.44, E08.49, E08.610, E09.40, E09.41, E09.42, E09.43, E09.44, E09.49, E09.610, E13.40, E13.41, E13.42, E13.43, E13.44, E13.49, E13.610, E08.51, E08.52, E08.59, E09.51, E09.52, E09.59, E13.51, E13.52, E13.59, E08.618, E08.620, E08.621, E08.622, E08.628, E08.630, E08.638, E08.649, E08.69, E09.618, E09.620, E09.621, E09.622, E09.628, E09.630, E09.638, E09.649, E09.69, E13.618, E13.620, E13.621, E13.622, E13.628, E13.630, E13.638, E13.649, E13.65, E13.69, E08.8, E09.8, E13.8, E11.9, E10.9, E11.65, E10.65, E11.10, E11.69, E10.10, E11.00, E11.01, E10.69, E11.11, E11.641, E10.11, E10.641, E11.21, E11.22, E11.29, E10.21, E10.22, E10.29, E11.311, E11.319, E11.3211, E11.3212, E11.3213, E11.3219, E11.3291, E11.3292, E11.3293, E11.3299, E11.3311, E11.3312, E11.3313, E11.3319, E11.3391, E11.3392, E11.3393, E11.3399, E11.3411, E11.3412, E11.3413, E11.3419, E11.3491, E11.3492, E11.3493, E11.3499, E11.3511, E11.3512, E11.3513, E11.3519, E11.3521, E11.3522, E11.3523, E11.3529, E11.3531, E11.3532, E11.3533, E11.3539, E11.3541, E11.3542, E11.3543, E11.3549, E11.3551, E11.3552, E11.3553, E11.3559, E11.3591, E11.3592, E11.3593, E11.3599, E11.36, E11.37X1, E11.37X2, E11.37X3, E11.37X9, E11.39, E10.311, E10.319, E10.3211, E10.3212, E10.3213, E10.3219, E10.3291, E10.3292, E10.3293, E10.3299, E10.3311, E10.3312, E10.3313, E10.3319, E10.3391, E10.3392, E10.3393, E10.3399, E10.3411, E10.3412, E10.3413, E10.3419, E10.3491, E10.3492, E10.3493, E10.3499, E10.3511, E10.3512, E10.3513, E10.3519, E10.3521, E10.3522, E10.3523, E10.3529, E10.3531, E10.3532, E10.3533, E10.3539, E10.3541, E10.3542, E10.3543, E10.3549, E10.3551, E10.3552, E10.3553, E10.3559, E10.3591, E10.3592, E10.3593, E10.3599, E10.36, E10.37X1, E10.37X2, E10.37X3, E10.37X9, E10.39, E11.40, E11.41, E11.42, E11.43, E11.44, E11.49, E11.610, E10.40, E10.41, E10.42, E10.43, E10.44, E10.49, E10.610, E11.51, E11.52, E11.59, E10.51, E10.52, E10.59, E11.618, E11.620, E11.621, E11.622, E11.628, E11.630, E11.638, E11.649, E10.618, E10.620, E10.621, E10.622, E10.628, E10.630, E10.638, E10.649, E11.8, E10.8
Diseases of other endocrine glands		

	251-255, 258, 259.8, 628.1	E15., E08.649, E16.0, E16.1, E16.2, E89.1, E16.3, E16.4, E16.8, E16.9, E21.3, E21.0, E21.1, E21.2, E20.0, E20.8, E20.9, E89.2, E21.4, E21.5, E22.0, E34.4, E22.1, E22.8, E22.9, E23.0, E23.6, E23.2, E22.2, E23.1, E89.3, E24.1, E23.3, E23.7, E32.0, E32.1, E32.8, E32.9, E24.0, E24.2, E24.3, E24.4, E24.8, E24.9, E26.09, E26.9, E26.02, E26.01, E26.81, E26.1, E26.89, E25.0, E25.8, E25.9, E27.0, E27.1, E27.2, E27.3, E27.40, E27.49, E89.6, E27.5, E27.8, E35., E27.9, E31.21, E31.22, E31.23, E31.0, E31.1, E31.20, E31.8, E31.9
Ovarian dysfunction		
	256	E28.0, E28.1, N98.1, E89.40, E89.41, E28.310, E28.319, E28.39, E28.2, E28.8, E28.9
Testicular dysfunction		
	257	E29.0, E89.5, E29.1, E29.8, E29.9
Nutritional deficiencies		
	260-269	E40., E42., E41., E43., E44.0, E44.1, E45., E46., E64.0, E50.0, E50.1, E50.2, E50.3, E50.4, E50.5, E50.6, E50.7, E50.8, E50.9, E64.1, E51.11, E51.12, E51.2, E51.8, E51.9, E52., E53.0, E53.1, D81.818, D81.819, E53.8, E53.9, E54., E64.2, E55.0, E64.3, M83.0, M83.1, M83.2, M83.3, M83.4, M83.5, M83.8, M83.9, E55.9, E56.1, E56.0, E56.8, E56.9, E58., E59., E60., E61.0, E61.1, E61.2, E61.3, E61.4, E61.5, E61.6, E61.7, E61.8, E63.0, E63.1, E63.8, E64.8, E61.9, E63.9, E64.9
Other metabolic diseases		
	270-279	E72.00, E72.01, E72.02, E72.04, E72.09, E70.0, E70.1, E70.20, E70.21, E70.29, E70.30, E70.310, E70.311, E70.318, E70.319, E70.320, E70.321, E70.328, E70.329, E70.330, E70.331, E70.338, E70.339, E70.39, E70.5, E70.8, E70.9, E71.0, E71.110, E71.111, E71.118, E71.120, E71.121, E71.128, E71.19, E71.2, E72.10, E72.11, E72.12, E72.19, E70.40, E70.41, E70.49, E72.20, E72.21, E72.22, E72.23, E72.29, E72.4, E72.3, E72.50, E72.51, E72.59, E72.8, E72.03, E72.9, E74.00, E74.01, E74.02, E74.03, E74.04, E74.09, E74.4, E74.20, E74.21, E74.29, E74.10, E74.11, E74.12, E74.19, E73.0, E73.1, E73.8, E73.9, E74.31, E74.39, E74.8, E72.52, E72.53, E77.1, E74.9, E78.00, E78.01, E78.1, E78.2, E78.3, E78.4, E78.5, E78.6, E88.1, E75.21, E75.22, E75.240, E75.241, E75.242, E75.243, E75.248, E75.249, E75.3, E77.0, E77.8, E77.9, E71.30, E75.5, E78.79, E78.81, E78.89, E88.2, E88.89, E75.6, E78.70, E78.9, D89.0, D47.2, D89.2, D89.1, C88.0, E88.01, E88.09, M10.00, M10.011, M10.012, M10.019, M10.021, M10.022, M10.029, M10.031, M10.032, M10.039, M10.041, M10.042, M10.049, M10.051, M10.052, M10.059, M10.061, M10.062, M10.069, M10.071, M10.072, M10.079, M10.08, M10.09, M10.10, M10.111, M10.112, M10.119, M10.121, M10.122, M10.129, M10.131, M10.132, M10.139, M10.141, M10.142, M10.149, M10.151, M10.152, M10.159, M10.161, M10.162, M10.169, M10.171, M10.172, M10.179, M10.18, M10.19, M10.20, M10.211, M10.212, M10.219, M10.221, M10.222, M10.229, M10.231, M10.232, M10.239, M10.241, M10.242, M10.249, M10.251, M10.252, M10.259, M10.261, M10.262, M10.269, M10.271, M10.272, M10.279, M10.28, M10.29, M1A.00X0, M1A.0110, M1A.0120, M1A.0190, M1A.0210, M1A.0220, M1A.0290, M1A.0310, M1A.0320, M1A.0390, M1A.0410, M1A.0420, M1A.0490, M1A.0510, M1A.0520, M1A.0590, M1A.0610, M1A.0620, M1A.0690, M1A.0710, M1A.0720, M1A.0790, M1A.08X0, M1A.09X0, M1A.20X0, M1A.2110, M1A.2120, M1A.2190, M1A.2210, M1A.2220, M1A.2290, M1A.2310, M1A.2320, M1A.2390, M1A.2410, M1A.2420, M1A.2490, M1A.2510, M1A.2520, M1A.2590, M1A.2610, M1A.2620, M1A.2690, M1A.2710, M1A.2720, M1A.2790, M1A.28X0, M1A.29X0, M1A.30X0, M1A.3110, M1A.3120, M1A.3190, M1A.3210, M1A.3220, M1A.3290, M1A.3310, M1A.3320, M1A.3390, M1A.3410, M1A.3420, M1A.3490, M1A.3510, M1A.3520, M1A.3590, M1A.3610, M1A.3620, M1A.3690, M1A.3710, M1A.3720, M1A.3790, M1A.38X0, M1A.39X0, M1A.40X0, M1A.4110, M1A.4120, M1A.4190, M1A.4210, M1A.4220, M1A.4290, M1A.4310, M1A.4320, M1A.4390, M1A.4410, M1A.4420, M1A.4490, M1A.4510, M1A.4520, M1A.4590, M1A.4610, M1A.4620, M1A.4690, M1A.4710, M1A.4720, M1A.4790, M1A.48X0, M1A.49X0, M1A.9XX0, M1A.00X1, M1A.0111, M1A.0121, M1A.0191, M1A.0211, M1A.0221, M1A.0291, M1A.0311, M1A.0321,

		M1A.0391, M1A.0411, M1A.0421, M1A.0491, M1A.0511, M1A.0521, M1A.0591, M1A.0611, M1A.0621, M1A.0691, M1A.0711, M1A.0721, M1A.0791, M1A.08X1, M1A.09X1, M1A.20X1, M1A.2111, M1A.2121, M1A.2191, M1A.2211, M1A.2221, M1A.2291, M1A.2311, M1A.2321, M1A.2391, M1A.2411, M1A.2421, M1A.2491, M1A.2511, M1A.2521, M1A.2591, M1A.2611, M1A.2621, M1A.2691, M1A.2711, M1A.2721, M1A.2791, M1A.28X1, M1A.29X1, M1A.30X1, M1A.3111, M1A.3121, M1A.3191, M1A.3211, M1A.3221, M1A.3291, M1A.3311, M1A.3321, M1A.3391, M1A.3411, M1A.3421, M1A.3491, M1A.3511, M1A.3521, M1A.3591, M1A.3611, M1A.3621, M1A.3691, M1A.3711, M1A.3721, M1A.3791, M1A.38X1, M1A.39X1, M1A.40X1, M1A.4111, M1A.4121, M1A.4191, M1A.4211, M1A.4221, M1A.4291, M1A.4311, M1A.4321, M1A.4391, M1A.4411, M1A.4421, M1A.4491, M1A.4511, M1A.4521, M1A.4591, M1A.4611, M1A.4621, M1A.4691, M1A.4711, M1A.4721, M1A.4791, M1A.48X1, M1A.49X1, M1A.9XX1, M10.30, M10.311, M10.312, M10.319, M10.321, M10.322, M10.329, M10.331, M10.332, M10.339, M10.341, M10.342, M10.349, M10.351, M10.352, M10.359, M10.361, M10.362, M10.369, M10.371, M10.372, M10.379, M10.38, M10.39, N20.0, M10.9, M10.40, M10.411, M10.412, M10.419, M10.421, M10.422, M10.429, M10.431, M10.432, M10.439, M10.441, M10.442, M10.449, M10.451, M10.452, M10.459, M10.461, M10.462, M10.469, M10.471, M10.472, M10.479, M10.48, M10.49, E83.110, E83.111, E83.118, E83.119, E83.10, E83.19, E83.00, E83.01, E83.09, E83.40, E83.41, E83.42, E83.49, E83.30, E83.31, E83.32, E83.39, E83.50, E83.51, E83.52, E20.1, E83.59, E83.81, E83.89, E83.9, E87.0, E87.1, E87.2, E87.3, E87.4, E86.9, E86.0, E86.1, E87.71, E87.70, E87.79, E87.5, E87.6, E87.8, E84.9, E84.11, E84.0, E84.19, E84.8, E80.0, E80.1, E80.20, E80.21, E80.29, D81.3, D81.5, E79.1, E79.2, E79.8, E79.9, E85.9, E85.0, M04.1, E85.1, E85.2, E85.3, E85.4, E85.81, E85.82, E85.89, E80.4, E80.5, E80.6, E80.7, E76.01, E76.02, E76.03, E76.1, E76.210, E76.211, E76.219, E76.22, E76.29, E76.3, E76.8, E76.9, D81.810, D84.1, E88.81, E71.41, E71.42, E71.43, E71.40, E71.440, E71.448, E71.310, E71.311, E71.312, E71.313, E71.314, E71.318, E71.32, E71.50, E71.510, E71.511, E71.518, E71.520, E71.521, E71.522, E71.528, E71.529, E71.53, E71.540, E71.541, E71.542, E71.548, E88.40, E88.41, E88.42, E88.49, H49.811, H49.812, H49.813, H49.819, E88.3, C96.5, C96.6, E71.39, E80.3, E88.9, E66.09, E66.1, E66.8, E66.9, E66.01, E66.3, E66.2, E65., E67.0, E67.1, E67.3, E67.2, E67.8, E68., D80.1, D80.2, D80.4, D80.3, D80.0, D80.5, D83.0, D83.1, D83.2, D83.8, D83.9, D80.6, D80.7, D80.8, D80.9, D84.8, D82.1, D82.0, D81.4, D81.0, D81.1, D81.2, D81.6, D81.7, D81.89, D81.9, D84.9, D89.82, D89.89, D89.813, D89.810, D89.811, D89.812, D82.2, D82.3, D82.4, D82.8, D82.9, D84.0, D89.3, D89.40, D89.41, D89.42, D89.43, D89.49, M35.9, D89.9
Avascular necrosis	733.4, 731.8	M87.00, M87.10, M87.20, M87.30, M87.80, M87.9, M90.50, M87.011, M87.012, M87.019, M87.021, M87.022, M87.029, M87.121, M87.122, M87.129, M87.221, M87.222, M87.229, M87.321, M87.322, M87.329, M87.821, M87.822, M87.829, M90.511, M90.512, M90.519, M87.051, M87.052, M87.059, M87.150, M87.151, M87.152, M87.159, M87.251, M87.252, M87.256, M87.350, M87.351, M87.352, M87.353, M87.850, M87.851, M87.852, M87.859, M90.551, M90.552, M90.559, M87.074, M87.075, M87.076, M87.174, M87.175, M87.176, M87.274, M87.275, M87.276, M87.374, M87.375, M87.376, M87.874, M87.875, M87.876, M87.08, M87.180, M87.031, M87.032, M87.033, M87.034, M87.035, M87.036, M87.037, M87.038, M87.039, M87.041, M87.042, M87.043, M87.044, M87.045, M87.046, M87.050, M87.061, M87.062, M87.063, M87.064, M87.065, M87.066, M87.071, M87.072, M87.073, M87.077, M87.078, M87.079, M87.09, M87.111, M87.112, M87.119, M87.131, M87.132, M87.133, M87.134, M87.135, M87.136, M87.137, M87.138, M87.139, M87.141, M87.142, M87.143, M87.144, M87.145, M87.146, M87.161, M87.162, M87.163, M87.164, M87.165, M87.166, M87.171, M87.172, M87.173, M87.177, M87.178, M87.179, M87.188, M87.19, M87.211, M87.212, M87.219, M87.231, M87.232, M87.233, M87.234, M87.235, M87.236, M87.237, M87.238, M87.239, M87.241, M87.242, M87.243, M87.244, M87.245, M87.246, M87.250, M87.261, M87.262, M87.263, M87.264, M87.265, M87.266, M87.271, M87.272, M87.273, M87.277, M87.278, M87.279, M87.28, M87.29, M87.311, M87.312, M87.319, M87.331, M87.332, M87.333, M87.334, M87.335, M87.336, M87.337, M87.338, M87.339, M87.341, M87.342, M87.343, M87.344, M87.345,

M87.346, M87.361, M87.362, M87.363, M87.364, M87.365, M87.366, M87.371, M87.372, M87.373, M87.377, M87.378, M87.379, M87.38, M87.39, M87.811, M87.812, M87.819, M87.831, M87.832, M87.833, M87.834, M87.835, M87.836, M87.837, M87.838, M87.839, M87.841, M87.842, M87.843, M87.844, M87.845, M87.849, M87.861, M87.862, M87.863, M87.864, M87.865, M87.869, M87.871, M87.872, M87.873, M87.877, M87.878, M87.879, M87.88, M87.89, M90.521, M90.522, M90.529, M90.531, M90.532, M90.539, M90.541, M90.542, M90.549, M90.561, M90.562, M90.569, M90.571, M90.572, M90.579, M90.58, M90.59

Supplementary Table 2. Secondary Neoplasms Reported >3 Years Following Diagnosis Among AYAs (ages 15-39) With ALL, 1995-2012, California, N=1,069*

	N	%
Second Cancers Reported	19	1.8
Lip		
Salivary Gland		
Soft Tissue		
Melanoma		
Other Non-Epithelial Skin		
Breast		
Vulva		
Testis		
Thyroid		
Hodgkin Lymphoma		
Miscellaneous		

* Unable to report cancer frequencies occurring in less than 5 individuals
AYA, adolescent and young adult; ALL, acute lymphoblastic leukemia

Supplementary Table 3. Ten-year cumulative incidence (%) with 95% CI of medical conditions among 3-year AYA ALL survivors, California, 1995-2012 for patients who entered the treatment algorithm (N=455) and for those who did not enter the treatment algorithm (N=614)

	Cardiac diseases	Seizure/Stroke	Respiratory system diseases	Renal disease	Liver disease	Endocrine disease	Avascular events	Second Cancers
	% (95% CI)	% (95% CI)	% (95% CI)	% (95% CI)	% (95% CI)	% (95% CI)	% (95% CI)	% (95% CI)
**Patients who entered treatment algorithm (N=455)**								
Front-line regimen								
Adult	14.9% (11.0% , 19.4%)	4.1% (2.2% , 7.0%)	3.7% (1.9% , 6.4%)	1.9% (0.7% , 4.1%)	3.7% (1.9% , 6.5%)	23.0% (18.2% , 28.1%)	6.9% (4.3% , 10.3%)	1.2% (0.3% , 3.1%)
Peds	10.6% (5.4% , 17.9%)	1.1% (0.1% , 5.2%)	5.3% (2.0% , 11.2%)	3.2% (0.8% , 8.3%)	2.1% (0.4% , 6.8%)	17.1% (10.2% , 25.4%)	8.5% (4.0% , 15.3%)	0.0% (0.0% , 0.0%)
P value	0.03	0.158	0.782	0.812	0.043	0.078	0.37	0.202
**Patients who did not enter treatment algorithm (N=614)**								
Front-line regimen								
Adult	21.0% (16.0% , 26.5%)	4.9% (2.7% , 8.2%)	8.9% (5.7% , 13.0%)	4.7% (2.6% , 7.7%)	8.3% (5.1% , 12.5%)	38.2% (32.0% , 44.3%)	9.2% (6.1% , 13.1%)	3.4% (1.4% , 6.7%)
Peds	18.2% (13.4% , 23.7%)	4.9% (2.5% , 8.3%)	6.7% (4.0% , 10.3%)	3.7% (1.7% , 6.9%)	8.9% (5.7% , 13.0%)	29.8% (23.9% , 36.0%)	13.5% (9.2% , 18.7%)	0.4% (0.0% , 2.2%)
P value	0.536	0.673	0.64	0.239	0.328	0.078	0.172	0.086

Data for patients with unclear front-line regimen are not presented

p values reflect the difference in cumulative incidence of late effects by baseline characteristics and treatment over entire study period using Gray's K-sample test statistic

Supplementary Table 4. Ten-Year Cumulative Incidence (%) with 95% CI of Late Effects Among 3-year AYA ALL Survivors, California, 1995-2012

	Cardiac diseases	Seizure/ Stroke	Respiratory system diseases	Renal disease	Liver disease	Endocrine disease	Avascular events	Second Cancers
	% (95% CI)	% (95% CI)	% (95% CI)	% (95% CI)	% (95% CI)	% (95% CI)	% (95% CI)	% (95% CI)
Race/ Ethnicity								
Asian/PI	11.6 (6.1,19.1)	3.5 (0.9,9.3)	5.1 (1.9,10.9)	1.1 (0.1,5.4)	4.1 (1.3,9.5)	21.4 (13.7,30.2)	7.5 (3.2,14.1)	1.0 (0.1,5.0)
Hispanic	20.0 (16.3, 23.9)	4.6 (2.9,6.9)	6.4 (4.4, 8.9)	4.0 (2.4, 6.2)	10.3 (7.6,13.4)	33.2 (28.8,37.6)	10.4 (7.7,13.4)	1.2 (0.4,2.7)
NH Black/ Other/Unknown	7.1 (1.8, 17.7)	2.4 (0.2,11.0)	4.7 (0.8,14.1)	*	5.6 (0.9,17.0)	23.4 (11.2,38.2)	8.2 (2.0, 20.4)	*
NH White	15.6 (11.9,19.8)	4.5 (2.6, 7.1)	6.4 (4.1, 9.4)	2.9 (1.5, 5.0)	2.4 (1.1,4.5)	25.2 (20.7,30.0)	9.3 (6.5,12.7)	1.7 (0.7,3.8)
P value	0.137	0.956	0.694	0.335	0.002	0.096	0.672	0.656
Age Group								
15-19	16.2 (12.8, 20.0)	3.7 (2.2, 5.9)	6.4 (4.3, 9.1)	3.4 (2.0, 5.6)	7.3 (5.1, 10.1)	26.3 (22.1,30.7)	11.3 (8.5,14.6)	1.2 (0.4,2.8)
20-29	16.4 (12.4, 21.0)	5.4 (3.1,8.8)	5.4 (3.2, 8.4)	2.6 (1.2, 4.9)	8.7 (5.7, 12.6)	31.5 (26.2,37.0)	8.3 (5.5,11.8)	1.1 (0.3, 3.1)
30-39	19.2 (14.1, 24.9)	4.1 (2.0, 7.4)	7.1 (4.1,11.1)	3.2 (1.4, 6.1)	2.3 (0.8, 4.9)	29.4 (23.4,35.6)	7.8 (4.7, 12.0)	2.0 (0.7, 4.9)
P value	0.131	0.451	0.751	0.316	0.043	0.12	0.308	0.373
Sex								
Female	18.3 (14.3, 22.6)	3.1 (1.6, 5.3)	7.1 (4.7, 10.1)	2.1 (1.0, 4.0)	6.4 (4.1, 9.4)	29.1 (24.4,34.0)	10.2 (7.4, 13.6)	1.2 (0.4, 2.9)
Male	16.2 (13.3, 19.4)	5.1 (3.5, 7.2)	5.7 (4.0, 7.8)	3.8 (2.4, 5.6)	6.6 (4.8, 8.9)	28.3 (24.7,32.1)	9.1 (6.9, 11.8)	1.4 (0.6, 2.9)
P value	0.634	0.213	0.616	0.841	0.77	0.707	0.405	0.385
Health Insurance								
Private/Military	14.9 (12.0, 18.2)	3.6 (2.2, 5.5)	5.4 (3.7, 7.6)	2.8 (1.6, 4.5)	3.9 (2.4, 5.9)	24.7 (21.1,28.4)	9.0 (6.7, 11.7)	2.1 (1.1, 3.7)
Public/ Uninsured	20.5 (16.3, 25.0)	5.8 (3.6, 8.8)	7.7 (5.2, 10.8)	3.5 (1.9, 5.9)	11.4 (8.2, 15.1)	36.6 (31.4,41.8)	10.9 (7.9, 14.4)	0.0 (0.0, 0.0)
P value	0.038	0.539	0.134	0.535	<0.0001	0.002	0.361	0.032
HCT								
No	10.4 (8.2, 12.8)	2.8 (1.7, 4.3)	2.6 (1.6, 4.0)	1.1 (0.5, 2.1)	4.8 (3.4, 6.6)	19.1 (16.2,22.2)	8.4 (6.5, 10.7)	0.3 (0.1, 1.2)
Yes	33.8 (27.9, 39.8)	8.2 (5.2, 12.1)	15.5(11.3,20)	8.2 (5.2, 12.1)	11.2 (7.5, 15.8)	53.2 (46.7,59.2)	12.5 (8.7, 16.9)	4.0 (1.9, 7.2)
P value	<0.0001	<0.0001	<0.0001	<0.0001	<0.0001	<0.0001	0.066	<0.0001
Neighborhood SES tertile								
Low	20.0 (15.9, 24.4)	6.2 (4.0, 9.1)	6.0 (3.9, 8.8)	3.2 (1.7, 5.5)	9.0 (6.2, 12.4)	34.3 (29.4,39.3)	9.3 (6.6, 12.7)	0.8 (0.2, 2.3)
Medium	15.6 (11.8, 19.9)	4.1 (2.2, 6.9)	8.2 (5.5, 11.7)	3.8 (2.1, 6.4)	6.4 (4.0, 9.5)	25.8 (21.1,30.8)	9.2 (6.3, 12.8)	2.2 (0.9, 4.6)
High	14.4 (10.4, 19.1)	2.1 (0.8, 4.6)	4.0 (2.0, 7.0)	2.2 (0.9, 4.6)	3.5 (1.7, 6.3)	24.3 (19.2,29.9)	10.3 (6.9, 14.4)	0.9 (0.2, 3.0)
P value	0.095	0.086	0.579	0.224	0.138	0.005	0.97	0.449
Treatment facility is COG or NCI CC								
Always	16.7 (13.5, 20.2)	3.8 (2.4, 5.8)	5.6 (3.9, 7.9)	3.3 (2.0, 5.1)	6.8 (4.8, 9.3)	28.0 (24.1,32.0)	10 (7.5,12.8)	1.0 (0.3, 2.4)

Partial/ None	17.0 (13.5, 20.8)	5.2 (3.3, 7.8)	6.7 (4.5, 9.5)	2.7 (1.4, 4.7)	6.0 (3.9, 8.6)	27.5 (23.2,31.9)	8.6 (6.1, 11.6)	1.6 (0.6, 3.3)
P value	0.526	0.511	0.85	0.476	0.81	0.003	0.31	0.573
Front-line regimen								
Adult	17.5 (14.4, 20.9)	4.5 (3.0, 6.6)	6.1 (4.3, 8.4)	3.3 (2.0, 5.1)	5.8 (4.0, 8.1)	30.1 (26.3,34.0)	8.0 (5.9, 10.4)	2.1 (1.1, 3.8)
Pediatrics	15.8 (12.0, 20.0)	3.7 (2.0, 6.3)	6.4 (4.1, 9.4)	3.4 (1.8, 5.9)	7.1 (4.6, 10.3)	26.0 (21.3,31.0)	11.7 (8.4,15.5)	0.3 (0.0, 1.6)
P value	0.223	0.816	0.957	0.626	0.589	0.194	0.159	0.085
CRT								
No	17.2 (14.4, 20.1)	4.2 (2.8, 5.9)	6.4 (4.7, 8.4)	3.2 (2.0, 4.7)	6.5 (4.8, 8.6)	29.0 (25.6,32.4)	9.8 (7.7, 12.2)	1.7 (0.9, 3.1)
Yes	15.6 (10.9, 21.2)	3.9 (1.7, 7.6)	4.3 (2.0, 7.9)	2.6 (1.0, 5.6)	6.3 (3.4, 10.3)	26.7 (20.6,33.1)	8.3 (5.0, 12.8)	0.5 (0.0, 2.6)
Unknown	20.8 (9.5, 35.1)	7.6 (1.9, 18.7)	12.9 (4.6,25.7)	5.0 (0.9, 14.9)	8.4 (2.0, 20.9)	33.1 (18.8,48.1)	13.0 (4.6, 25.8)	0.0 (0.0,0.00)
P value	0.98	0.64	0.189	0.744	0.881	0.461	0.718	0.05

CI, confidence interval; AYA, adolescent and young adult; ALL, acute lymphoblastic leukemia; PI, Pacific Islander; NH, non-Hispanic; HCT, hematopoietic cell transplant; SES, socioeconomic status; CRT, cranial irradiation therapy
*Small number of events prevented estimation of cumulative incidence in this category.
